# TBA-MLR score: a metabolic-immune prognostic biomarker for postoperative hepatocellular carcinoma

**DOI:** 10.3389/fimmu.2025.1628571

**Published:** 2025-09-05

**Authors:** Yuanquan Zhao, Wei Huang, Xiwen Deng, Pengyang Li, Siyang Yao, Yongyu Yang, Xiaofeng Dong

**Affiliations:** ^1^ Department of Hepatobiliary, Pancreas and Spleen Surgery, The People’s Hospital of Guangxi Zhuang Autonomous Region, Nanning, China; ^2^ Department of Emergency, The People’s Hospital of Guangxi Zhuang Autonomous Region, Nanning, China

**Keywords:** hepatocellular carcinoma, total bile acid, monocyte-to-lymphocyte ratio, prognostic score, radical hepatectomy

## Abstract

**Objective:**

To develop and validate a novel prognostic score combining serum total bile acid (TBA) and monocyte-to-lymphocyte ratio (MLR) for improved risk stratification in hepatocellular carcinoma (HCC) patients after radical hepatectomy.

**Methods:**

In 508 HCC patients undergoing radical hepatectomy, we determined optimal TBA and MLR cutoffs for RFS and OS using maximally selected rank statistics. Multivariable Cox regression identified independent predictors, enabling development of a three-tiered TBA-MLR risk score (low/intermediate/high). We internally validated performance via bootstrapping (1000 iterations) and compared it against conventional biomarkers (AFP, BCLC, Child-Pugh) and inflammatory indices (SII, SIRI, NLR, PLR). Subgroup analyses assessed its ability to refine prognosis within BCLC stages and AFP categories. Concordance and overlap were assessed via Venn diagrams and Cohen’s kappa coefficient. Subgroup analyses assessed the robustness of the TBA-MLR score.

**Results:**

Elevated TBA (>11.7 μmol/L; HR=2.96, p<0.001) and MLR (>0.26; HR=1.64, p=0.001) independently predicted poorer RFS, while TBA (>14 μmol/L; HR=3.87, p<0.001) and MLR (>0.32; HR=1.54, p=0.036) were associated with worse OS. The TBA-MLR score stratified patients into distinct risk groups: high-risk patients had significantly lower 1/3/5-year RFS (66.9%/41.4%/19.1%) and OS (79.5%/51.1%/19.1%) versus low-risk patients (RFS:94.3%/80.8%/73.8%; OS:97.9%/90.8%/85.3%; HR=5.69 and 4.07, both p<0.001). Notably, it identified high-risk subsets within traditional low-risk categories: 22.7% of BCLC0-A patients were high-risk by TBA-MLR and had a 5-year OS of only 22.6% (vs. 86.2% in low-risk BCLC0-A patients, p<0.0001). Similarly, among patients with AFP <400 ng/mL, the high-risk group (21.9%) had a 5-year OS of 31.9% (vs. 82.7% in low-risk patients, p<0.0001). Internal validation confirmed strong predictive accuracy (C-indices: RFS 0.639, OS 0.683), with 1/3/5-year AUCs (RFS:0.657/0.660/0.771; OS:0.713/0.720/0.779) outperforming conventional biomarkers (all p<0.05). The score demonstrated minimal concordance with conventional systems (|κ|<0.06), with 16–25% of high-risk patients missed by BCLC/AFP criteria. Subgroup analyses showed consistent performance across tumor characteristics and treatments.

**Conclusion:**

The TBA-MLR score is a robust metabolic-immune prognostic biomarker that effectively uncovers occult high-risk biology within conventional staging systems, enabling precise postoperative risk stratification for individualized management, particularly for patients traditionally classified as low-risk (e.g., BCLC 0-A) or with non-elevated AFP.

## Introduction

Hepatocellular carcinoma (HCC), the most common primary liver cancer, accounts for 75–85% of global liver cancer cases. Its incidence has risen steadily over the past two decades, with pronounced geographic clustering in Asia and hepatitis B virus (HBV)-endemic regions (1). Although surgical advances have established radical hepatectomy as the gold standard for early-stage HCC, postoperative recurrence rates remain alarmingly high (50–70% at five years), severely limiting long-term survival (2, 3). Consequently, accurate risk stratification for recurrence and mortality is critical for optimizing personalized follow-up and adjuvant therapies. This clinical challenge underscores the necessity for refined prognostic tools; identifying high-risk patients early could enable tailored interventions (e.g., targeted or immunotherapies) and intensified surveillance to improve outcomes.

Current prognostic systems like BCLC and TNM staging rely heavily on anatomical features (tumor size, vascular invasion) and liver function (Child-Pugh score). However, their predictive accuracy remains suboptimal (4, 5). For instance, BCLC stage A patients exhibit >3-fold survival variability, highlighting these systems’ inability to account for tumor biological heterogeneity (6). While alpha-fetoprotein (AFP) serves as a serological biomarker, its limitations are well-documented: low sensitivity (approximately 40% of HCC patients exhibit normal AFP) and susceptibility to non-tumor confounders (e.g., liver regeneration, pregnancy) (7). Therefore, such rigid staging approaches fall short of precision medicine demands for HCC management.

Emerging evidence links metabolic dysfunction and immune microenvironment disruption as pivotal drivers of HCC progression (8, 9). Serum total bile acid (TBA), a central regulator of hepatic metabolism, acts not only as a liver dysfunction marker but also directly promotes invasiveness by activating farnesoid X receptor (FXR) and G protein-coupled bile acid receptor 5 (TGR5) signaling (10, 11). Preclinical studies reveal TBA’s dual role in tumorigenesis: inducing DNA damage via mitochondrial reactive oxygen species (ROS) to accelerate genomic instability and upregulating PD-L1 to promote immune evasion (12, 13). Clinically, elevated preoperative TBA correlates with poor liver function and aggressive tumor biology, supporting its prognostic utility (14). Collectively, these findings provide a solid theoretical foundation for TBA as a predictive biomarker.

Conversely, systemic inflammation plays a dual role in HCC development: activating carcinogenic pathways via pro-inflammatory cytokines (e.g., IL-6, TNF-α) and fostering immunosuppression through cells like M2 macrophages and myeloid-derived suppressor cells (15–17). The monocyte-to-lymphocyte ratio (MLR), an emerging immune balance indicator, demonstrates prognostic value in various solid tumors (18–20). Monocytes facilitate tumor angiogenesis and extracellular matrix remodeling by secreting vascular endothelial growth factor (VEGF) and matrix metalloproteinases (MMPs), while lymphocyte depletion (particularly cytotoxic T-cells) impairs immune surveillance (21). Significantly, bile acids can polarize monocytes toward an immunosuppressive phenotype via TGR5 activation, suggesting potential TBA-MLR crosstalk (22).

Although TBA and MLR possess independent prognostic value, no study has systematically evaluated their combined effects. This approach is biologically plausible: TBA-represented metabolic abnormalities and MLR-reflected inflammation may synergize via “metabolic-immunological crosstalk” to shape a pro-tumor microenvironment. Practically, both markers are routinely accessible through standard blood tests, ensuring clinical feasibility. Our study addresses this gap by developing the TBA-MLR score, with optimal cutoffs determined via maximally selected rank statistics and rigorously validated through bootstrap resampling (n = 1000). Through comprehensive comparisons and subgroup analyses, we demonstrate this score’s superiority over conventional biomarkers for precise risk stratification. These findings inform novel strategies for postoperative monitoring and personalized HCC therapy.

## Materials and methods

### Study design and patient cohort

This retrospective cohort study enrolled HCC patients who underwent radical hepatectomy at the People’s Hospital of Guangxi Zhuang Autonomous Region (Guangxi Academy of Medical Science) between January 2015 and December 2021. Radical resection was defined as pathologically confirmed R0 resection (negative margins) with preoperative imaging and intraoperative exploration showing no extrahepatic metastasis or major vascular invasion. Inclusion criteria: (1) age ≥18 years; (2) first hepatectomy without other primary liver tumors; (3) postoperative pathological diagnosis of HCC; (4) complete preoperative laboratory data and postoperative follow-up records. Exclusion criteria included: (1) concomitant history of other malignancies; (2) perioperative mortality; (3) preoperative radiotherapy/chemotherapy/targeted therapy; (4) loss to follow-up or incomplete data; (5) concurrent bile acid metabolism disorders (e.g., choledocholithiasis, cholangitis) or severe extrahepatic diseases (e.g., Child-Pugh class C cirrhosis, active autoimmune/infectious diseases). Our study ultimately enrolled 508 patients and exempted them from informed consent. The institutional ethics committee approved the study protocol.

### Data collection and variable definitions

Fasting venous blood samples collected within 48 hours preoperatively were analyzed for serum TBA and inflammatory indices: MLR, platelet-to-lymphocyte ratio (PLR), neutrophil-to-lymphocyte ratio (NLR), systemic inflammation response index (SIRI = neutrophil × monocyte/lymphocyte), and systemic immune-inflammation index (SII = neutrophil × platelet/lymphocyte). Clinicopathological variables included: Demographics (age, sex, body mass index [BMI]); Laboratory parameters (alanine aminotransferase [ALT], aspartate aminotransferase [AST], total bilirubin [TBIL], albumin [ALB], platelet count [PLT], C-reactive protein [CRP], alpha-fetoprotein [AFP], carcinoembryonic antigen [CEA]); Virological status (HBsAg positivity defined HBV infection; HBV-DNA load stratified by 500 IU/mL cutoff); Tumor characteristics (maximum diameter, number, differentiation, microvascular invasion [MVI], hepatic capsule invasion, BCLC stage, Child-Pugh grade). Adjuvant therapies, including transarterial chemoembolization (TACE), were documented. Targeted therapies (e.g., sorafenib) and immunotherapies (e.g., anti-PD-1) were not administered postoperatively in this cohort, as they were not standard during the study period (2015–2021). Postoperative follow-up included tri-monthly abdominal ultrasonography, contrast-enhanced CT/MRI, and serum AFP testing. The researchers recorded recurrence patterns as: intrahepatic recurrence (IHR), pulmonary metastasis (PM), and non-pulmonary extrahepatic metastasis (NP-EHM). We defined recurrence-free survival (RFS) as the time from surgery to radiologically confirmed recurrence or last follow-up. Overall survival (OS) spanned from surgery to all-cause death or last follow-up (censored June 2023).

### Variable processing and categorization

We categorized continuous variables per clinical guidelines or prior standards: age (≤60 vs. >60 years), AFP (≤200/200–1000/>1000 ng/mL), and tumor size (≤5 cm vs. >5cm). For inflammatory indices, we applied established cutoffs to dichotomize SIRI ≥0.785×10^9^/L (23), SII ≥600×10^9^/L (24), PLR ≥98.89 (25), and NLR ≥2.50 (26). We also converted BMI, CRP, PLT, ALT, AST, TBIL, ALB, and CEA to binary/tertiary variables based on clinical reference ranges.

### Statistical analysis

We expressed continuous variables as mean ± SD or median (IQR) and categorical variables as frequencies (%). Maximally selected rank statistics determined optimal TBA and MLR cutoffs for RFS/OS prediction. Univariate Cox regression (p<0.05) screened potential prognostic factors; significant variables underwent multivariate Cox regression to identify independent predictors. The TBA-MLR score was constructed by dichotomizing TBA and MLR (above cutoff = 1, below = 0), yielding a 0–2 score stratifying patients into low- (0), intermediate- (1), and high-risk (2) groups. Kaplan-Meier survival analysis and log-rank tests assessed prognostic performance versus established systems (BCLC, AFP). In addition, we used Venn diagrams and Cohen’s kappa coefficient to analyze the concordance and overlap between the TBA-MLR score and existing systems.

The score’s internal validation employed bootstrap resampling (1000 iterations). Assessment of its performance utilized the concordance index (C-index) and time-dependent receiver operating characteristic (ROC) area under the curve (AUC). We also evaluated prognostic superiority over conventional indicators (AFP, BCLC, Child-Pugh) and inflammatory markers (SII, SIRI, NLR, PLR) via ROC curve comparison (DeLong test). Subgroup analyses stratified by tumor size (≤5 cm vs. >5cm), MVI status, AFP level (≤400 vs. >400 ng/mL), HBV infection, cirrhosis background, and postoperative adjuvant TACE validated robustness. All analyses used R 4.2.2 (packages: maxstat, survival, timeROC). Two-tailed p<0.05 indicated statistical significance.

### Rationale for biomarker selection

The TBA-MLR score captures synergistic metabolic-immune interactions implicated in HCC progression. Serum TBA (a regulator of bile acid signaling) and MLR (an indicator of monocyte-driven inflammation) were prioritized based on: (i) preclinical evidence of their crosstalk in fostering immunosuppressive microenvironments (10, 22, 27); (ii) clinical feasibility as widely accessible blood biomarkers; and (iii) superior discriminatory power over tumor-burden variables (e.g., AFP, MVI) in preliminary time-dependent ROC analyses. While other factors (AFP, MVI) showed prognostic significance, their inclusion would not enhance the score’s mechanistic focus and could reduce clinical utility for preoperative risk assessment.

## Results

### Baseline characteristics of study participants

**Table 1** summarizes the baseline characteristics of the 508 patients. The cohort was predominantly male (81.3%), with a median age of 53.0 years (IQR 44.0–63.0) and a BMI of 23.44 kg/m² (IQR 21.35–25.80). Hematological analysis revealed median values of 3.59 ×10^9^/L (IQR 2.62–4.64) for neutrophils, 1.81 ×10^9^/L (IQR 1.43–2.24) for lymphocytes, and 0.55 ×10^9^/L (IQR 0.39–0.72) for monocytes, yielding a median MLR of 0.30 (IQR 0.22–0.41). Key metabolic parameters included TBA (8.70 μmol/L; IQR 4.30–15.83), ALT (32.00 U/L; IQR 22.00–50.25), AST (35.50 U/L; IQR 26.00–53.25), and AFP (60.80 ng/mL; IQR 6.42–783.89). Clinically, 37.6% (n = 191) had cirrhosis, and 70.9% (n = 360) were HBsAg positive. Notably, 63.4% (n = 322) had HBV-DNA levels below 500 IU/mL, and 92.3% (n = 469) were Child-Pugh class A. Clinicians administered postoperative adjuvant TACE to 22.4% (n = 114). Tumor characteristics indicated solitary lesions in 86.0% (n = 437), a median diameter of 4.60cm (IQR 2.80–6.70), and microvascular invasion in 34.1% (n = 173). Most patients (82.1%, n = 417) were BCLC stage A. During a median follow-up of 24.4 months (IQR 12.47–37.90), tumor recurrence occurred in 44.3% (n = 225). Among recurrences, IHR was most common (n = 154, 30.3% of cohort), followed by PM (n = 40, 7.9%) and NP-EHM (n = 31, 6.1%). The cohort’s median overall survival was 26.7 months (IQR 14.20-46.62), with cumulative mortality reaching 22.0% (n = 112).

**Table 1:** Baseline characteristics of the study population.

Characteristic	Overall (n=508)
Demographics	
Gender, n (%)	
Female	95 (18.7%)
Male	413 (81.3%)
Age (years), median [IQR]	53.00 [44.00, 63.00]
BMI (kg/m^2^), median [IQR]	23.44 [21.35, 25.80]
Laboratory parameters	
CRP (mg/L), median [IQR]	7.08 [1.23, 40.14]
Neutrophil count (×10^9^/L), median [IQR]	3.59 [2.62, 4.64]
Lymphocyte count (×10^9^/L), median [IQR]	1.81 [1.43, 2.24]
Monocyte count (×10^9^/L), median [IQR]	0.55 [0.39, 0.72]
MLR, median [IQR]	0.30 [0.22, 0.41]
PLT (×10^9^/L), median [IQR]	196.00 [143.50, 250.25]
TBA (µmol/L), median [IQR]	8.70 [4.30, 15.83]
ALT (U/L), median [IQR]	32.00 [22.00, 50.25]
AST (U/L), median [IQR]	35.50 [26.00, 53.25]
TBIL (µmol/L), median [IQR]	13.30 [10.20, 17.70]
ALB (g/L), median [IQR]	38.20 [35.08, 40.90]
AFP (ng/mL), median [IQR]	60.80 [6.42, 783.89]
CEA (ng/mL), median [IQR]	2.04 [1.32, 2.86]
Clinical features	
Cirrhosis, n (%)	
no	317 (62.4%)
yes	191 (37.6%)
HBsAg, n (%)	
negative	148 (29.1%)
positive	360 (70.9%)
HBV-DNA, n (%)	
<500IU/mL	322 (63.4%)
≥500IU/mL	186 (36.6%)
Child-Pugh class, n (%)	
A	469 (92.3%)
B	39 (7.7%)
Tumor number, n (%)	
single	437 (86.0%)
multiple	71 (14.0%)
Tumor size (cm), median [IQR]	4.60 [2.80, 6.70]
MVI, n (%)	
no	335 (65.9%)
yes	173 (34.1%)
Differentiation, n (%)	
Poorly differentiated	39 (7.7%)
Moderately differentiated	413 (81.3%)
Well-differentiated	56 (11.0%)
Hepatic capsule invasion, n (%)	
no	400 (78.7%)
yes	108 (21.3%)
BCLC stage, n (%)	
0	41 (8.1%)
A	417 (82.1%)
B	50 (9.8%)
TACE, n (%)	
no	394 (77.6%)
yes	114 (22.4%)
Outcomes	
Recurrence, n (%)	225 (44.3%)
Recurrence sites, n (%)	
IHR	154 (30.3%)
PM	40 (7.9%)
NP-EHM	31 (6.1%)
NR	283 (55.7%)
RFS (months), median [IQR]	24.4 [12.5, 37.9]
Survival, n (%)	112 (22.0%)
OS (months), median [IQR]	26.7 [14.2, 46.6]

BMI, body mass index; CRP, C-reactive protein; MLR, monocyte-to-lymphocyte ratio; PLT, platelet count; TBA, total bile acid; ALT, alanine aminotransferase; AST, aspartate aminotransferase; TBIL, total bilirubin; ALB, albumin; AFP, alpha-fetoprotein; CEA, carcinoembryonic antigen; HBsAg, hepatitis B surface antigen; MVI, microvascular invasion; BCLC, Barcelona Clinic Liver Cancer; TACE, Transcatheter Arterial Chemoembolization; IHR, Intrahepatic Recurrence; PM, Pulmonary Metastasis; NP-EHM, Non-Pulmonary Extrahepatic Metastases; NR, No Recurrence; RFS, recurrence-free survival; OS, overall survival.

Data are presented as median [interquartile range (IQR)] or n (%).

### Prognostic significance of serum TBA and MLR

**Figure 1:**
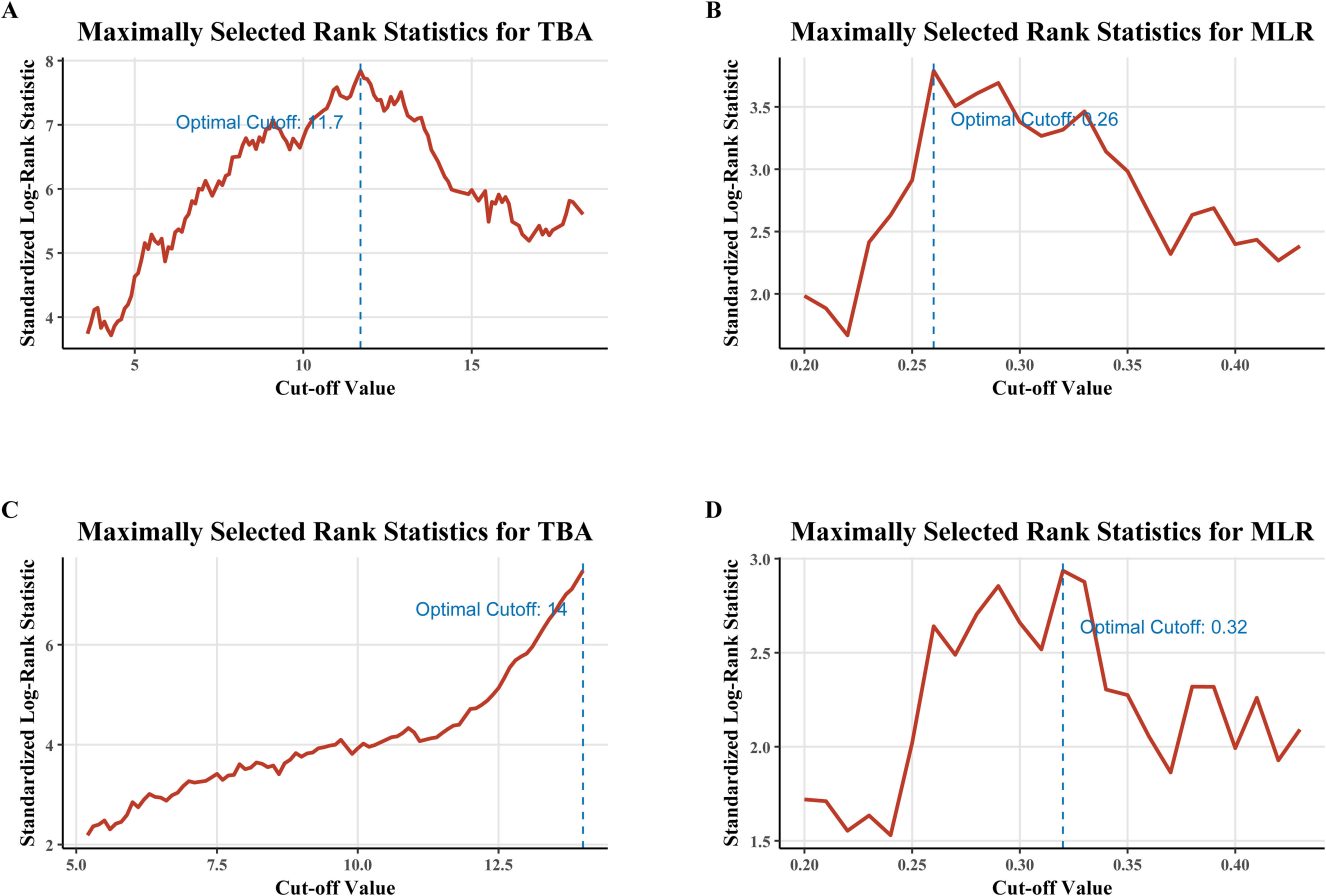
Determination of optimal cutoff values for TBA and MLR using maximally selected rank statistics. **(A)** TBA cutoff (11.7 μmol/L) for recurrence-free survival (RFS). **(B)** MLR cutoff (0.26) for RFS. **(C)** TBA cutoff (14 μmol/L) for overall survival (OS). **(D)** MLR cutoff (0.32) for OS. Cutoffs were derived from log-rank statistics to maximize between-group survival differences.

**Figure 2:**
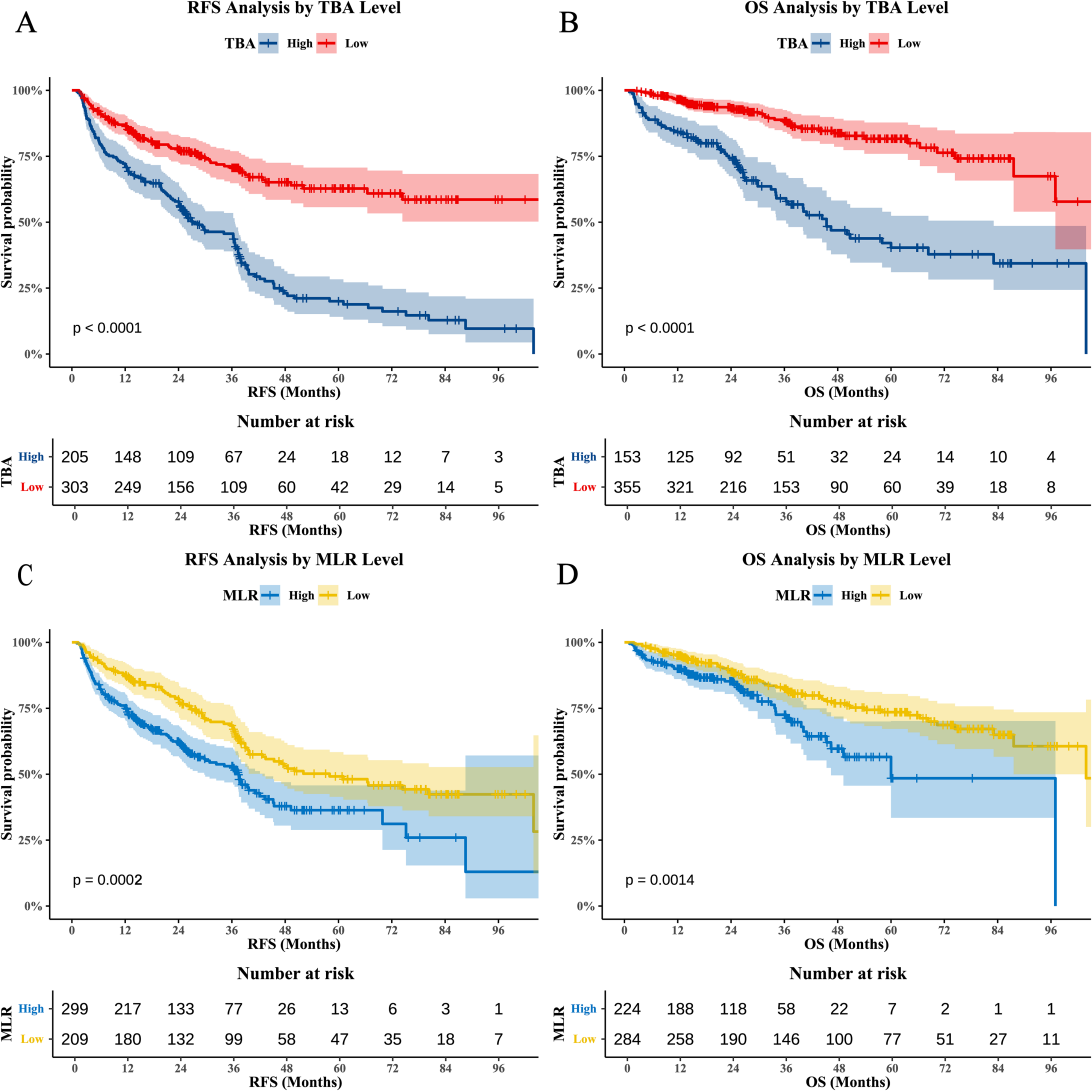
Kaplan-Meier survival curves for TBA and MLR. **(A)** RFS stratified by TBA (BAati vs. >11.7 μmol/L; log-rank p<0.0001). **(B)** OS stratified by TBA (BAa vs. >14 μmol/L; log-rank p<0.0001). **(C)** RFS stratified by MLR (LRati vs. >0.26; log-rank p=0.0002). **(D)** OS stratified by MLR (LRati vs. >0.32; log-rank p=0.0014).

Maximally selected rank statistics identified optimal TBA and MLR cutoffs for risk stratification (**Figure 1**). TBA thresholds were 11.7 μmol/L for RFS (**Figure 1A**) and 14 μmol/L for OS (**Figure 1C**), while MLR cutoffs were 0.26 (RFS; **Figure 1B**) and 0.32 (OS; **Figure 1D**), demonstrating the dynamic differences in risk stratification thresholds for biomarkers across clinical endpoints. Kaplan-Meier analyses confirmed independent prognostic value: high TBA (p<0.0001) and MLR (p=0.0002) groups exhibited significantly reduced RFS (**Figures 2A, C**), while OS was similarly compromised (high TBA: p<0.0001; high MLR: p=0.0014; **Figures 2B, D**).

### Independent prognostic factors for RFS and OS

Univariate and multivariate Cox regression analyses confirmed the independent predictive value of serum TBA and MLR for post-hepatectomy recurrence and mortality in HCC patients (**Tables 2**, **3**).

**Table 2:** Univariate and multivariate Cox regression analysis of recurrence-free survival.

Variable	Univariate Analysis		Multivariate Analysis	
	HR 95%CI	*P*-value	HR 95%CI	*P*-value
Gender (male)	1.25 (0.876–1.773)	0.222	–	–
Age (<60 years)	1.50 (1.124–2.008)	**0.006**	1.58 (1.161–2.15)	**0.004**
BMI (kg/m^2^)				
<18.5 vs 18.5–24	0.92 (0.506–1.663)	0.776	–	–
<18.5 vs ≥24	1.11 (0.610–2.030)	0.728	–	–
CRP (RP2 mg/L)	0.89 (0.684–1.159)	0.387	–	–
PLT (×10^9^/L)				
<100 vs 100–300	1.20 (0.761–1.880)	0.437	–	–
<100 vs >300	0.84 (0.456–1.553)	0.581	–	–
ALT (LT8 U/L)	1.08 (0.791–1.460)	0.644	–	–
AST (ST4 U/L)	0.83 (0.637–1.084)	0.172	–	–
TBIL (BIL27 µmol/L)	1.00 (0.746–1.340)	0.998	–	–
ALB (LB9 g/L)	1.00 (0.728–1.369)	0.993	–	–
AFP (ng/mL)				
≤200 vs 200–1000	1.80 (1.366–2.362)	**<0.001**	1.47 (1.104–1.968)	**0.009**
≤200 vs>1000	1.53 (0.894–2.629)	0.121	2.28 (1.290–4.015)	**0.005**
CEA (>5 ng/mL)	2.11 (1.261–3.531)	**0.004**	1.94 (1.137–3.324)	**0.015**
Cirrhosis (yes)	1.09 (0.824–1.435)	0.555	–	–
HBsAg (positive)	1.17 (0.869–1.570)	0.303	–	–
HBV-DNA (NA03 IU/mL)	1.36 (1.042–1.772)	**0.024**	1.07 (0.809–1.417)	0.633
Child-Pugh class (B)	1.47 (0.904–2.385)	0.121		
Tumor number (multiple)	1.82 (1.278–2.593)	**0.001**	1.49 (0.746–2.960)	0.259
Tumor size (iz cm)	1.86 (1.432–2.424)	**<0.001**	1.27 (0.895–1.788)	0.184
MVI (yes)	2.39 (1.822–3.123)	**<0.001**	1.73 (1.071–2.787)	**0.025**
Differentiation				
PD vs MD	0.84 (0.537–1.327)	0.463	–	–
PD vs WD	0.57 (0.313–1.051)	0.072	–	–
Hepatic capsule invasion (yes)	1.42 (1.049–1.922)	**0.023**	1.25 (0.917–1.704)	0.159
BCLC				
0 vs A	1.78 (0.968–3.270)	0.063	1.31 (0.686–2.508)	0.413
0 vs B	3.49 (1.732–7.027)	**<0.001**	1.01 (0.363–2.809)	0.985
TACE (yes)	3.10 (2.327–4.116)	**<0.001**	1.33 (0.751–2.338)	0.332
TBA (>11.7 µmol/L)	2.87 (2.187–3.769)	**<0.001**	2.96 (2.229–3.917)	**<0.001**
MLR (>0.26)	1.69 (1.286–2.230)	**<0.001**	1.64 (1.230–2.196)	**0.001**

BMI, body mass index; CRP, C-reactive protein; MLR, monocyte-to-lymphocyte ratio; PLT, platelet count; TBA, total bile acid; ALT, alanine aminotransferase; AST, aspartate aminotransferase; TBIL, total bilirubin; ALB, albumin; AFP, alpha-fetoprotein; CEA, carcinoembryonic antigen; HBsAg, hepatitis B surface antigen; MVI, microvascular invasion; PD, Poorly differentiated; MD, Moderately differentiated; WD, Well-differentiated; BCLC, Barcelona Clinic Liver Cancer; TACE, Transcatheter Arterial Chemoembolization; HR, hazard ratio; CI, confidence interval.

Bold values denote a p-value < 0.05, indicating statistical significance.

**Table 3:** Univariate and multivariate Cox regression analysis of overall survival.

Variable	Univariate Analysis		Multivariate Analysis	
	HR 95% CI	*P-*value	HR 95% CI	*P-*value
Gender (male)	1.46 (0.862–2.486)	0.159	–	–
Age (<60 years)	1.37 (0.911–2.054)	0.131	–	–
BMI (kg/m^2^)				
<18.5 vs 18.5–24	0.75 (0.357–1.554)	0.433	–	–
<18.5 vs ≥s8	0.62 (0.290–1.336)	0.224	–	–
CRP (RP2 mg/L)	0.82 (0.560–1.188)	0.288	–	–
PLT (×10^9^/L)				
<100 vs 100–300	1.14 (0.592–2.191)	0.698	–	–
<100 vs >300	1.05 (0.454–2.433)	0.908	–	–
ALT (LT0 U/L)	0.94 (0.612–1.431)	0.759	–	–
AST (ST5 U/L)	0.67 (0.456–0.969)	**0.034**	1.05 (0.701–1.558)	0.830
TBIL (BIL01 µmol/L)	1.05 (0.688–1.591)	0.833	–	–
ALB (LB3 g/L)	0.81 (0.526–1.244)	0.334	–	–
AFP (ng/mL)				
≤ng/vs 200–1000	1.85 (1.244–2.746)	**0.002**	1.43 (0.941–2.176)	0.094
≤.09 vs>1000	2.77 (1.502–5.093)	**0.001**	3.13 (1.642–5.979)	**0.001**
CEA (>5 ng/mL)	1.18 (0.480–2.917)	0.714		
Cirrhosis (yes)	1.01 (0.675–1.515)	0.958	–	–
HBsAg (positive)	1.30 (0.848–1.996)	0.229	–	–
HBV-DNA (NA29 IU/mL)	1.30 (0.890–1.894)	0.175	–	–
Child-Pugh class (B)	1.61 (0.807–3.193)	0.178	–	–
Tumor number (multiple)	1.66 (0.984–2.803)	0.057	–	–
Tumor size (iz cm)	2.32 (1.584–3.408)	**<0.001**	1.44 (0.866–2.400)	0.159
MVI (yes)	3.36 (2.282–4.943)	**<0.001**	2.63 (1.295–5.332)	**0.007**
Differentiation				
PD vs MD	0.83 (0.454–1.516)	0.544	0.70 (0.373–1.300)	0.256
PD vs WD	0.39 (0.152–0.982)	**0.046**	0.54 (0.203–1.445)	0.221
Hepatic capsule invasion (yes)	1.07 (0.679–1.677)	0.777	–	–
BCLC				
0 vs A	4.78 (1.178–19.387)	**0.029**	2.90 (0.688–12.227)	0.147
0 vs B	8.31 (1.870–36.908)	**0.005**	3.66 (0.779–17.211)	0.100
TACE (yes)	4.10 (2.775–6.060)	**<0.001**	1.11 (0.492–2.510)	0.800
TBA (>14 µmol/L)	3.92 (2.683–5.734)	**<0.001**	3.87 (2.606–5.744)	**<0.001**
MLR (>0.32)	1.87 (1.267–2.769)	**0.002**	1.54 (1.023–2.312)	**0.039**

BMI, body mass index; CRP, C-reactive protein; MLR, monocyte-to-lymphocyte ratio; PLT, platelet count; TBA, total bile acid; ALT, alanine aminotransferase; AST, aspartate aminotransferase; TBIL, total bilirubin; ALB, albumin; AFP, alpha-fetoprotein; CEA, carcinoembryonic antigen; HBsAg, hepatitis B surface antigen; MVI, microvascular invasion; PD, Poorly differentiated; MD, Moderately differentiated; WD, Well-differentiated; BCLC, Barcelona Clinic Liver Cancer; TACE, Transcatheter Arterial Chemoembolization; HR, hazard ratio; CI, confidence interval.

Bold values denote a p-value < 0.05, indicating statistical significance.

The univariate analysis for RFS revealed two significant risk factors: elevated TBA (>11.7 µmol/L; HR=2.87, 95% CI 2.187–3.769, p<0.001) and increased MLR (>0.26; HR=1.69, 95% CI 1.286–2.230, p<0.001). These associations persisted after multivariate adjustment, with TBA (HR=2.96, 95% CI 2.229–3.917, p<0.001) and MLR (HR=1.64, 95% CI 1.230–2.196, p=0.001) remaining independent predictors. Other factors independently associated with poorer RFS included advanced age (>60 years; HR=1.58, p=0.004), elevated AFP (200–1000 ng/mL: HR=1.47, p=0.009; >1000 ng/mL: HR=2.28, p=0.005), high CEA (>5 ng/mL; HR=1.94, p=0.015), and presence of MVI (HR=1.73, p=0.025). In the OS analysis, univariate analysis identified TBA >14 µmol/L (HR=3.92, 95% CI 2.683–5.734, p<0.001) and MLR >0.32 (HR=1.87, 95% CI 1.267–2.769, p=0.002) as significant mortality risk factors. Multivariate analysis confirmed their independent prognostic value: TBA (HR=3.87, 95% CI 2.606–5.744, p<0.001) and MLR (HR=1.54, 95% CI 1.023–2.312, p=0.039). Notably, extremely high AFP (>1000 ng/mL; HR=3.13, p=0.001) and MVI (HR=2.63, p<0.001) showed powerful associations with mortality. However, our data showed that TACE itself was not significantly associated with RFS (HR=1.33, 95% CI 0.751–2.338, p=0.332) and OS (HR=1.11, 95% CI 0.492–2.510, p=0.800).

### Development and risk stratification of the TBA-MLR score system

As shown in **Table 4**, we developed a TBA-MLR score system based on the independent prognostic thresholds of preoperative serum TBA and MLR. The TBA-MLR scoring system defined TBA cutoff values as >11.7 μmol/L for RFS and >14 μmol/L for OS while setting MLR thresholds at >0.26 for RFS and >0.32 for OS. We stratified patients into three risk categories: low-risk (0 points)—patients exceeding neither TBA nor MLR thresholds; intermediate-risk (1 point)—patients exceeding either parameter’s threshold; and high-risk (2 points)—patients exceeding both parameters’ thresholds.

**Table 4:** TBA-MLR scoring systems for predicting RFS and OS after R0 hepatectomy in HCC.

Category	Criteria	TBA cutoff (RFS)	MLR cutoff (RFS)	TBA cutoff (OS)	MLR cutoff (OS)	Total score	Risk level
Scoring items	TBA elevation	>11.7 μmol/L	–	>14 μmol/L	–	+1	Intermediate/High
	MLR elevation	–	>0.26	–	>0.32	+1	Intermediate/High
Risk stratification							
Both parameters normal		≤11.7 μmol/L	≤0.26	≤14 μmol/L	≤0.32	0	Low risk
Either parameter elevated		>11.7 μmol/L *or*	>0.26 *or*	>14 μmol/L *or*	>0.32 *or*	1	Intermediate risk
Both parameters elevated		>11.7 μmol/L *and*	>0.26 *and*	>14 μmol/L *and*	>0.32 *and*	2	High risk

TBA, total bile acid; MLR, monocyte-to-lymphocyte ratio.

### Survival analysis of the TBA-MLR score

**Figure 3:**
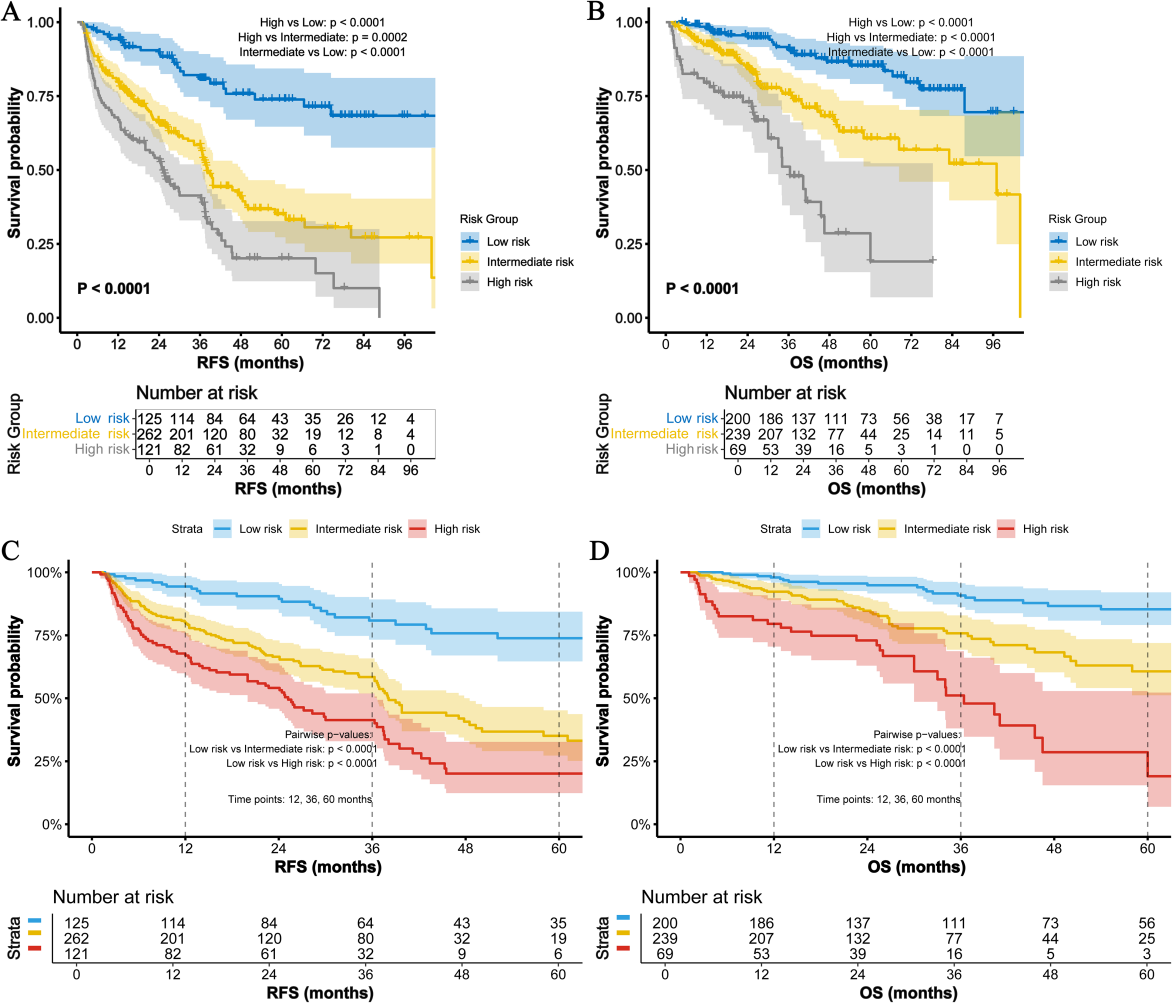
Survival analysis of the TBA-MLR score. **(A)** RFS and **(B)** OS for low- (0 points), intermediate- (1 point), and high-risk (2 points) groups. High-risk patients exhibited significantly worse outcomes (RFS: HR=5.69, 95% CI 3.61–8.95; OS: HR=4.07, 95% CI 2.24–7.41; all pairwise log-rank p<0.001). **(C)** 1-/3-/5-year RFS rates and **(D)** OS rates across risk groups.

Kaplan-Meier analysis (**Figures 3A–D**) demonstrated striking survival disparities among risk groups (all pairwise comparisons: p<0.0001). High-risk patients showed dramatically worse outcomes: 1-, 3-, and 5-year RFS rates were 66.9%, 41.4%, and 20.1%, respectively, versus 94.3%, 80.8%, and 73.8% in low-risk patients (HR=5.69, 95% CI 3.61–8.95, p < 0.0001). Intermediate-risk patients exhibited transitional results (79.9%/58.4%/35.1%; HR=3.37, 95% CI 2.18–5.20, p=0.0002). This pattern was more pronounced for OS. High-risk patients had substantially lower 1-/3-/5-year OS rates (79.5%/51.1%/19.1%) than low-risk counterparts (97.9%/90.8%/85.3%; HR=4.07, 95% CI 2.24–7.41, p<0.0001). Intermediate-risk patients again showed intermediate outcomes (92.3%/75.8%/60.7%; HR=2.52, 95% CI 1.42–4.47, p<0.0001).

### Refining prognostic stratification within conventional staging systems using the TBA-MLR score

To further delineate the complementary prognostic value of the TBA-MLR score, we performed a detailed stratification analysis within the conventional BCLC stage and AFP level categories (**Supplementary Table S1**). The distribution of TBA-MLR risk categories varied substantially within each group. For instance, among the 458 BCLC 0-A patients traditionally considered at lower risk, 22.7% (104/458) were classified as high-risk by the TBA-MLR score for RFS. Similarly, 13.3% (61/458) of BCLC 0-A patients were high-risk for OS. Critically, this heterogeneity was also pronounced within the AFP <400 ng/mL subgroup, which is often clinically challenging due to the lack of reliable prognostic markers. Here, 21.9% (76/347) and 11.8% (41/347) of patients were identified as high-risk for RFS and OS, respectively, by the TBA-MLR score (**Supplementary Table S1**).

The survival outcomes of these substratified groups revealed dramatic disparities obscured by conventional staging alone (**Supplementary Table S2**, **Supplementary Figure S1**). Notably, high-risk BCLC 0-A patients exhibited a median RFS of only 25.8 months and a 5-year OS rate of 22.6%, which were significantly worse than intermediate-risk and low-risk patients within the same BCLC stage (p<0.0001; **Supplementary Figures S1A, B**). This analysis highlights the score’s ability to identify a subset of patients with occult high-risk biology who are misclassified as low-risk by the BCLC system. The stratification within the AFP <400 ng/mL subgroup was particularly revealing (**Supplementary Figures S1C, D**). High-risk patients in this AFP “low” category suffered from poor outcomes, with a median RFS of 36.3 months and a 5-year RFS rate of only 29.0%. Their 5-year OS rate was also markedly reduced to 31.9%. Conversely, AFP <400 ng/mL patients who were also TBA-MLR low-risk had excellent outcomes, with a 5-year OS rate of 82.7% (HR for high- vs. low-risk = 4.07, p<0.001). Similarly, among patients with AFP ≥400 ng/mL, those also classified as high-risk by the TBA-MLR score had a median RFS of merely 7.8 months and a 5-year OS rate of 0%, starkly contrasting with the favorable outcomes of AFP-high patients who were TBA-MLR low-risk (median RFS not reached, 5-year OS 90.5%; p<0.0001; **Supplementary Figures S1C, D**).

These findings demonstrate that the TBA-MLR score provides critical prognostic refinement within all conventional categories, most notably by identifying a high-risk population among patients with normal or moderately elevated AFP levels (<400 ng/mL), a group for which current clinical guidance is least defined.

### Validation and comparative analysis of the TBA-MLR score

#### Internal validation

**Figure 4:**
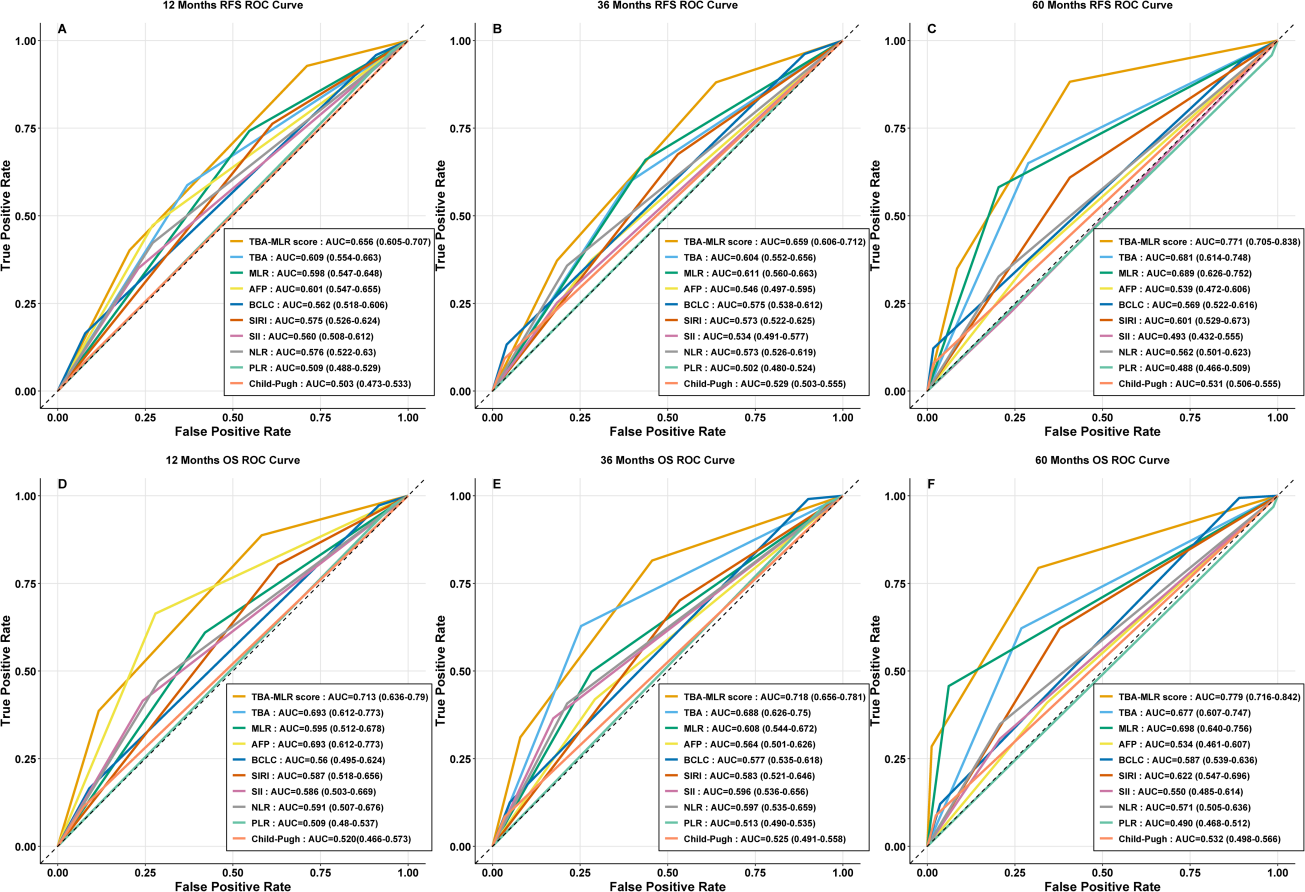
Time-dependent ROC curves for the TBA-MLR score. **(A–C)** RFS prediction at 1, 3, and 5 years (AUC: 0.656, 0.659, 0.771). **(D–F)** OS prediction at 1, 3, and 5 years (AUC: 0.713, 0.718, 0.779). Comparisons with single markers (TBA, MLR), conventional markers (AFP, BCLC, Child-Pugh), and inflammatory indices (SII, SIRI, NLR, PLR) are shown.

Internal validation through bootstrap resampling (1,000 iterations) confirmed the TBA-MLR score’s discriminative ability, yielding C-indices of 0.639 (95% CI 0.597–0.673) for RFS and 0.682 (95% CI 0.628–0.728) for OS. Time-dependent ROC curves (**Figures 4A–F**) consistently showed the composite score’s superiority over individual biomarkers (TBA, MLR), conventional prognostic factors (AFP, BCLC stage, Child-Pugh score), and established inflammatory indices (SII, SIRI, NLR, PLR) across all evaluated time points.

The TBA-MLR score demonstrated medium predictive accuracy for RFS, with AUC values reaching 0.656 (95% CI 0.605–0.707) at 1 year, 0.659 (95% CI 0.606–0.712) at 3 years, and 0.771 (95% CI 0.705–0.838) at 5 years. Its performance for OS prediction was similarly satisfactory, showing AUCs of 0.713 (95% CI 0.636–0.790), 0.718 (95% CI 0.656–0.781), and 0.779 (95% CI 0.716–0.842) at the same intervals. In contrast, all comparator metrics — including TBA or MLR alone (AUCs consistently below 0.700) — showed significantly poorer predictive capacity.

### Longitudinal trends and stability of the TBA-MLR score in prognostic prediction

**Figure 5:**
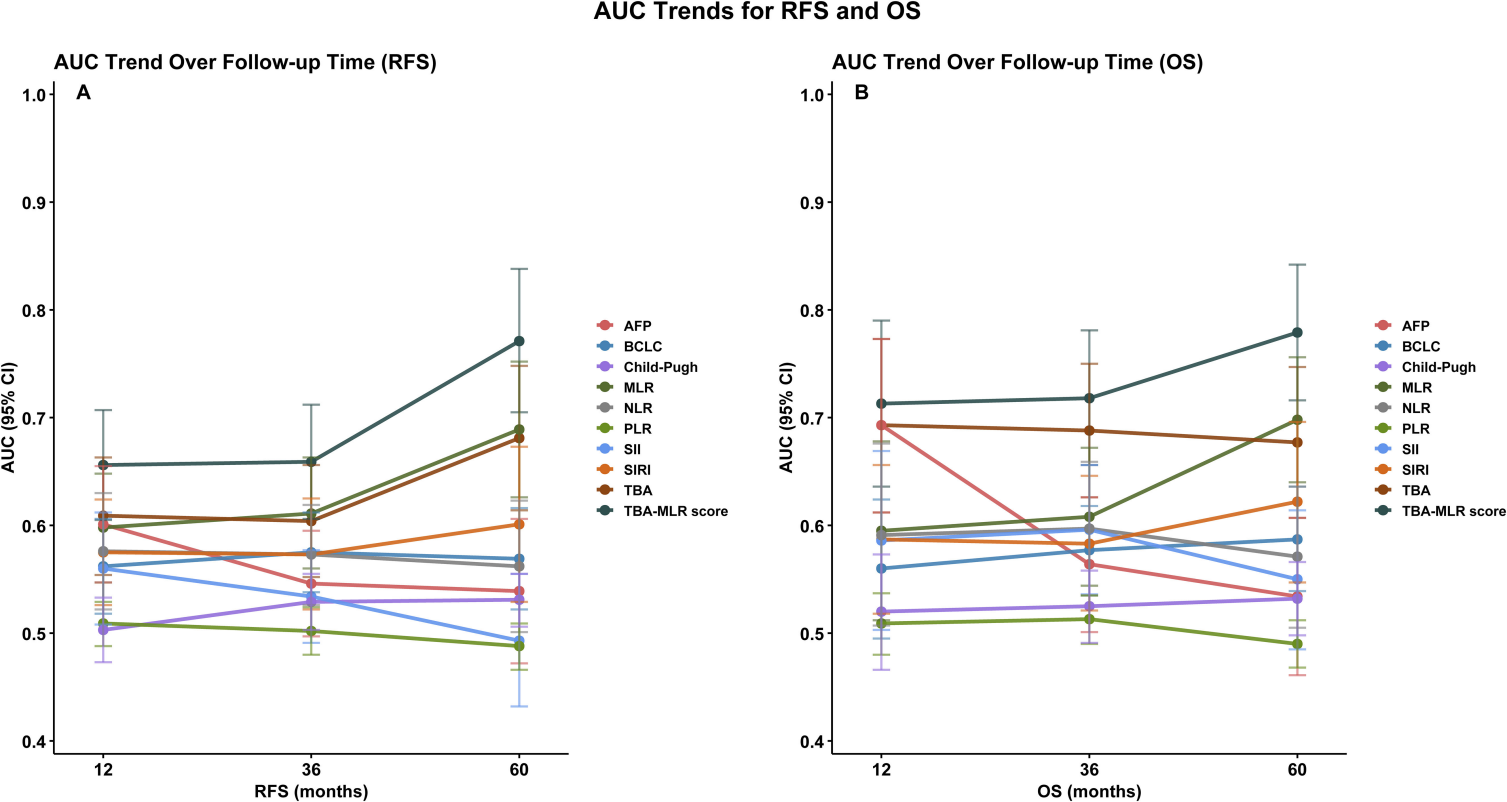
Longitudinal trends in the predictive accuracy of the TBA-MLR score. **(A)** RFS and **(B)** OS AUC values over time (1, 3, 5 years). The TBA-MLR score demonstrated increasing predictive accuracy (ΔAUC +0.115 for RFS,+0.066 for OS), superior to all comparable markers.

**Figure 6:**
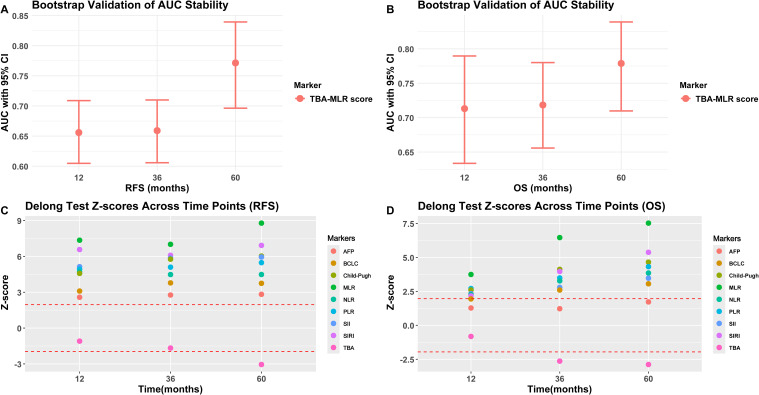
Bootstrap validation and DeLong test results. **(A, B)** Bootstrap-adjusted AUC for RFS and OS (95% CI). **(C, D)** DeLong’s test compares the TBA-MLR score and alternative biomarkers (showing Z scores and p-values predicted at 1, 3, and 5 years).

Longitudinal trend analysis (**Figures 5A, B**) demonstrated that the TBA-MLR score’s predictive accuracy improved consistently over time. For RFS, the AUC increased from 0.656 at 1 year to 0.771 at 5 years (ΔAUC +0.115), while OS prediction showed an AUC rise from 0.713 to 0.779 (ΔAUC +0.066). In contrast, other prognostic markers (AFP, BCLC, PLR, etc.) exhibited declining or inconsistent AUC trends during extended follow-up. Some conventional indicators (Child-Pugh, SII) performed poorly in long-term evaluations, with AUC values dropping below 0.550. Internal validation via bootstrap resampling confirmed the stability of the TBA-MLR score across all time points (**Figures 6A, B**). For RFS, the AUC remained robust, with values of 0.657 (95% CI: 0.605–0.709) at 1 year, 0.660 (0.606–0.710) at 3 years, and 0.771 (0.696–0.839) at 5 years. Similarly, OS prediction showed consistent performance, with AUCs of 0.713 (0.634–0.790), 0.719 (0.656–0.780), and 0.778 (0.710–0.839) at 1, 3, and 5 years, respectively. These results highlight the TBA-MLR score’s reliability and superior long-term prognostic capability compared to traditional markers.

### Statistical comparisons via the DeLong test

The DeLong’s test (**Figures 6C, D**) showed that the TBA-MLR score was significantly better than the single index (MLR), conventional parameters (BCLC, Child-Pugh), and inflammation index (SII, SIRI, NLR, PLR), all comparisons reaching statistical significance (|Z|>1.96, adjusted p<0.05). Its predictions at 5 years showed intense discrimination (e.g., TBA-MLR vs. MLR: RFS Z=8.79, adjusted p<0.001; OS Z=7.53, adjusted p<0.001). Interestingly, initial comparisons between the TBA-MLR score and isolated TBA showed no significant differences in early follow-up (1-year RFS: |Z| = 1.09, p=0.274; 3-year RFS: |Z| = 1.67, p=0.095; 1-year OS: |Z| = 0.82, p=0.414). However, with the extension of follow-up time, the cumulative prediction advantage of this score becomes obvious compared with TBA alone (5-year RFS: |Z| = 3.05, p=0.003; 3-year OS: |Z| = 2.64, p=0.010; 5-year OS: |Z| = 2.89, p=0.004), which is consistent with the bootstrap-validated AUC trends (**Figures 6A, B**). At all time points, the score was significantly better than AFP for RFS prediction (all p<0.05). Although the comparison between the OS prediction of this score and AFP is not statistically significant (for example, 5-year OS: |Z| = 1.72, p=0.085), the absolute AUC difference of 0.245 (0.779 vs. 0.534, **Figure 4E**) surpassed clinically relevant net benefit thresholds. These findings highlight the TBA-MLR score’s evolving predictive power over time, demonstrating superior robustness and clinical utility for long-term prognosis assessment. The complete DeLong’s test results for all time points are available in **Supplementary Tables S3–S4**.

### Comparative analysis of baseline characteristics across TBA-MLR risk groups

To address potential confounding effects, we conducted comprehensive comparisons of baseline characteristics among TBA-MLR risk strata (**Supplementary Table S5**). Significant differences were observed across groups, aligning with the pathophysiological basis of the score.

The analysis performed a comparative analysis of 21 baseline characteristics across 121 high-risk, 262 intermediate-risk, and 125 low-risk patients (for TBA-MLR score Risk Stratification for RFS). High-risk patients exhibited significantly poorer liver function: higher AST abnormality rates (49.6% vs. 41.2% vs. 32.0%, p=0.019), elevated TBIL levels (>17.1 μmol/L: 38.8% vs. 28.2% vs. 20.0%, p=0.005), and reduced ALB levels (<35 g/L: 34.7% vs. 23.3% vs. 16.8%, p=0.004). We observed more aggressive tumor features: higher MVI rates (39.7% vs. 35.9% vs. 24.8%, p=0.032) and increased multifocal tumor incidence (19.8% vs. 14.9% vs. 6.4%, p=0.008). Additionally, the high-risk group had higher cirrhosis prevalence (52.9% vs. 34.7% vs. 28.8%, p<0.001) and received adjuvant TACE more frequently (30.6% vs. 24.8% vs. 9.6%, p<0.001).

The baseline characteristics of 69 high-risk, 239 intermediate-risk, and 200 low-risk patients stratified by TBA-MLR score for OS showed similar trends. Liver function comparisons showed higher proportions with TBIL >17.1 μmol/L (42.0% vs. 33.1% vs. 19.0%, p<0.001) and ALB <35 g/L (33.3% vs. 28.5% vs. 16.5%, p=0.003). Tumor burden indicators revealed increased multifocal tumors (20.3% vs. 17.6% vs. 7.5%, p=0.003) and higher MVI rates (43.5% vs. 37.7% vs. 26.5%, p=0.010). Cirrhosis prevalence also increased significantly (55.1% vs. 38.5% vs. 30.5%, p=0.001) and received adjuvant TACE more frequently (31.9% vs. 27.2% vs. 13.5%, p<0.001).

### Subgroup validation of prognostic stratification

We performed multidimensional subgroup analyses to verify the prognostic value of the TBA-MLR score among key clinical variables: cirrhosis status, HBV infection, tumor size, microvascular invasion (MVI), and AFP levels. The scoring system demonstrated consistent predictive performance across all evaluated subgroups.

**Figure 7:**
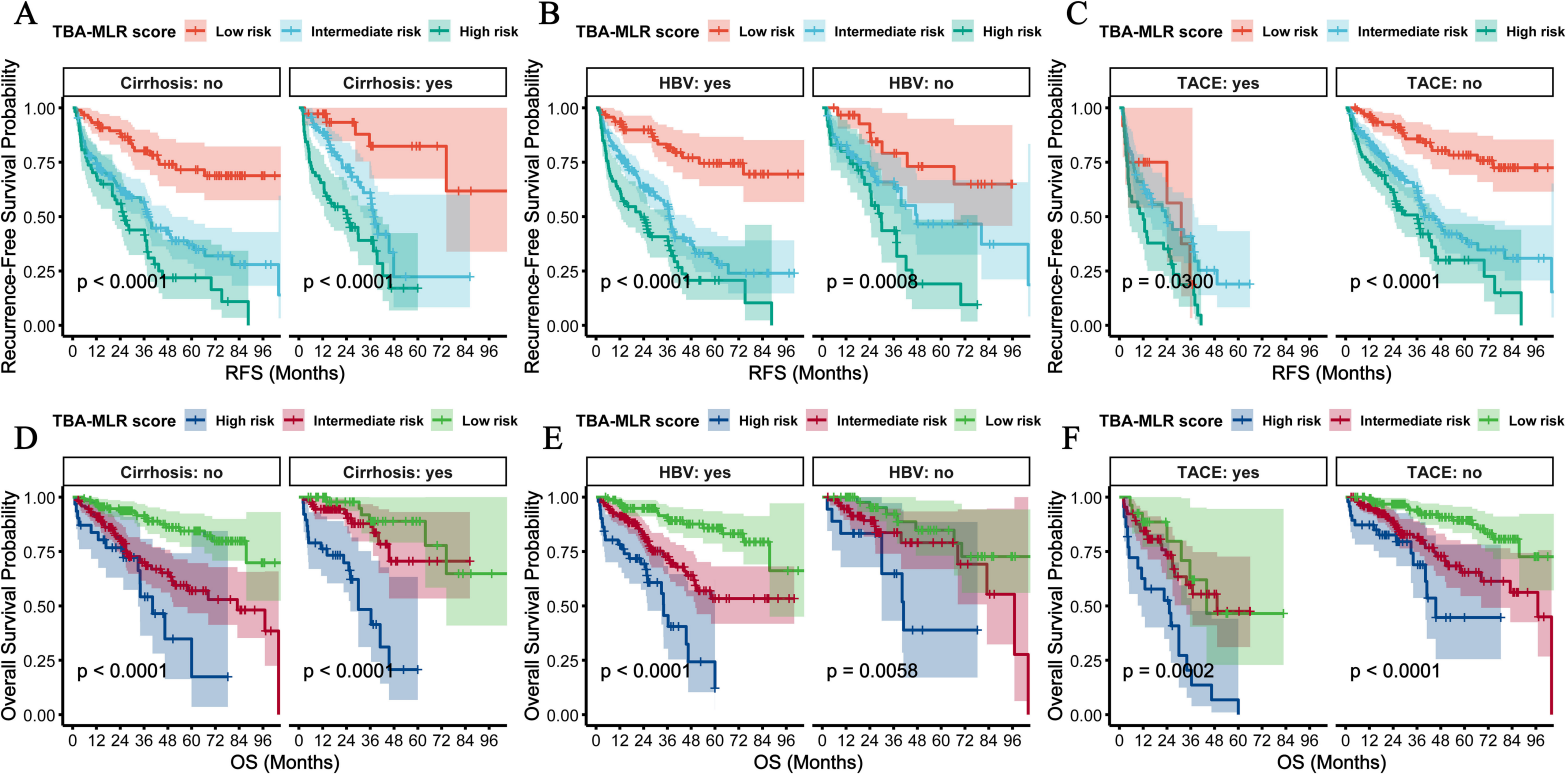
Subgroup analyses by HBV infection, cirrhosis status, and postoperative adjuvant TACE treatment. **(A)** RFS and **(C)** OS in cirrhosis and non-cirrhosis subgroups. High-risk patients consistently showed poorer survival (all log-rank p<0.0001). **(B, D)** Similar results were observed for HBV-positive vs. HBV-negative subgroups. **(C, D)** In the postoperative adjuvant TACE treatment subgroup, high-risk patients showed significantly worse RFS (TACE, p=0.0300; no TACE, p<0.001) and OS (TACE, p=0.0002; no TACE, p<0.001) regardless of TACE administration.

**Figure 8:**
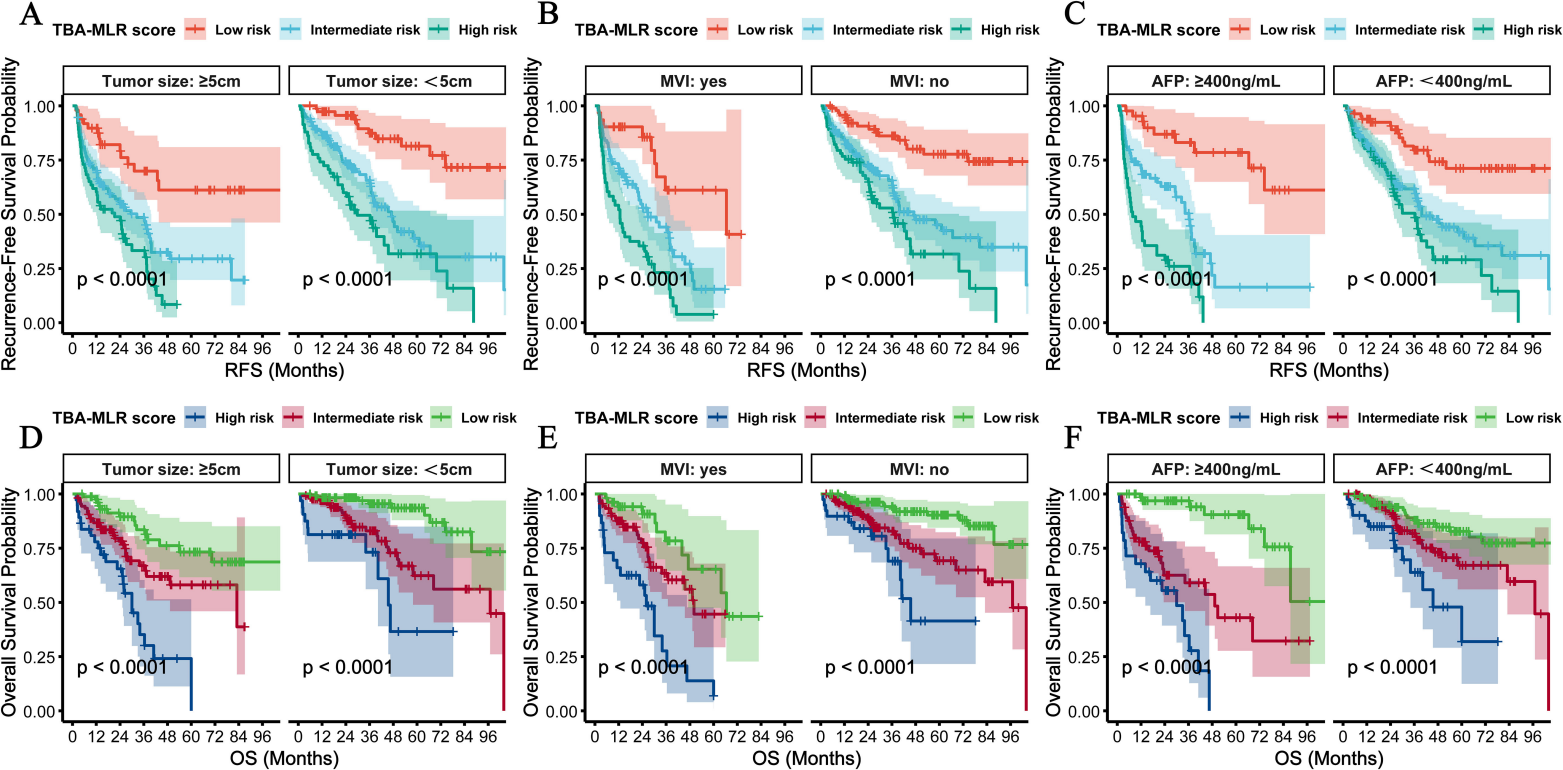
Subgroup analyses by tumor size, MVI, and AFP levels. **(A, B, D, E)** RFS and OS were stratified by tumor size (<5cm vs. ≥5 cm) and MVI status. **(C, F)** Prognostic performance of patients with <400 vs ≥sth ng/mL AFP levels. The TBA-MLR score maintained robust discrimination across all subgroups (log-rank p<0.0001).

Regardless of cirrhosis status, patients classified as high-risk showed significantly worse outcomes, with the cumulative RFS and OS differences reaching high statistical significance (p<0.0001, **Figures 7A, D**). Similar results occurred when stratified by HBV infection status, with high-risk patients exhibiting substantially worse cumulative RFS (p<0.0001) and OS (p<0.0001) compared to low-risk patients (**Figures 7B, E**). Notably, the stratification showed consistent results in the postoperative adjuvant TACE treatment subgroup: high-risk patients showed significantly worse RFS (TACE, p=0.0300; no-TACE, p<0.001) and OS (TACE, p=0.0002; no-TACE, p<0.001) regardless of TACE administration (**Figures 7C, F**). Adjuvant postoperative TACE failed to improve outcomes in high-risk patients (RFS: rapid decline to 0.4 at 24 months; OS: near zero at 48 months), while providing survival benefits only for low- to moderate-risk groups. Stratification of tumor size showed excellent prognostic value of this score, with high-risk patients showing significantly worse prognosis in the ≥5 cm and <5cm subgroups (cumulative RFS and OS: p<0.0001, **Figures 8A, D**). Similarly, the scoring system still maintained excellent discrimination under MVI status and AFP level stratification (> 400 vs <400 ng/mL), and all comparisons maintained statistical significance (cumulative RFS and OS: p<0.0001, **Figures 8B, C, E, F**). The multidimensional subgroup analyses confirmed that TBA-MLR scores provide reliable risk stratification across different HCC patient populations.

### Concordance analysis with existing staging systems

To assess the clinical differentiation of the TBA-MLR score from established prognostic systems, we performed Cohen’s kappa (κ) analysis and Venn diagram-based overlap quantification. As shown in **Supplementary Table S6**, the TBA-MLR score demonstrated negligible concordance with both BCLC staging (RFS: Weighted κ = 0.041, p=0.155; OS: Weighted κ = 0.020, p=0.438) and AFP stratification (RFS: κ = -0.037, p=0.146; OS: κ = -0.056, p=0.091), with all |κ|<0.06 indicating minimal agreement (Landis & Koch criteria). Notably, the Venn diagram (**Supplementary Figure S2**) illustrates that the TBA-MLR score identifies distinct high-risk biology beyond traditional systems. For RFS prognosis, 25% (65/257) of patients classified as high-risk by the TBA-MLR score were not categorized as high-risk by either BCLC-B stage or AFP ≥400 ng/mL. Similarly, for OS prognosis, 16% (36/228) of TBA-MLR high-risk patients were missed by these conventional criteria. Conversely, minimal overlap existed, with only 1–2% of patients classified as high-risk by all three definitions. This low concordance underscores the TBA-MLR score’s capacity to identify a unique high-risk patient subset not captured by BCLC-B stage or AFP levels alone.

## Discussion

This study is the first to propose and validate the prognostic value of the TBA-MLR scoring system, integrating serum TBA and MLR, for predicting recurrence and survival after radical hepatectomy in HCC. Our results demonstrate that the TBA-MLR score enables precise risk stratification, outperforming conventional biomarkers (AFP, BCLC stage, Child-Pugh score) and existing inflammatory indices (SII, SIRI, NLR, PLR), thereby providing a novel biological basis for personalized postoperative management.

It is noteworthy that the baseline comparison of risk stratification (**Supplementary Table S5**) confirmed that high-risk patients exhibited significantly worse metabolic-immune profiles—including higher cirrhosis prevalence (RFS: 52.9% vs 28.8%, p<0.001; OS: 55.1% vs 30.5%, p=0.001), elevated MVI rates (RFS: 39.7% vs 24.8%, p=0.032; OS: 43.5% vs 26.5%, p=0.010), and impaired liver function (e.g., hypoalbuminemia: RFS p=0.004, OS p=0.003). These differences follow an expected mechanism: Elevated TBA promotes fibrogenesis via FXR/TGR5-dependent stellate cell activation, accelerating cirrhosis (14, 28). MLR-driven inflammation facilitates EMT and microvascular invasion through MMP-9/VEGF secretion (21, 29). Thus, the observed baseline imbalance is not a confounder but rather an inherent validation of the score’s biological coherence in capturing aggressive HCC phenotypes. Moreover, our multivariate Cox models (**Tables 2**, **3**) and subgroup analyses (**Figures 7**, **8**) confirmed the score’s independent prognostic value after adjusting for confounders (e.g., MVI, tumor size, cirrhosis). Most importantly, our refined stratification analysis (**Supplementary Table S2**, **Supplementary Figure S1**) underscores TBA-MLR’s ability to uncover critical risk heterogeneity within conventional categories. For instance, the stark divergence in 5-year OS between high-risk and low-risk patients within BCLC stage A (22.6% vs. 86.2%) reveals profound biological aggression not captured by anatomical staging. This is of paramount clinical value, as it challenges the uniform “good prognosis” label assigned to all BCLC A patients and mandates a more personalized approach to postoperative surveillance and adjuvant therapy planning. Similarly, the score’s performance within the AFP <400 ng/mL subgroup addresses a significant clinical blind spot. Current guidelines offer limited risk stratification for this large patient subset in the absence of elevated AFP. Our findings demonstrate that the TBA-MLR score can effectively segment this group into distinct prognostic categories, with high-risk patients suffering a 5-year OS of only 31.9% compared to 82.7% in low-risk patients. This ability to identify high-risk biology independent of AFP level not only reinforces the score’s mechanistic basis in metabolic-immune pathways but also provides a much-needed tool for optimizing management in AFP-negative/low patients.

From a metabolic perspective, elevated TBA levels reflect hepatic dysfunction and may remodel the tumor microenvironment through bile acid receptor-mediated pathways (e.g., FXR, TGR5) (11, 28). Prior evidence indicates that bile acids promote tumor proliferation and angiogenesis via FXR-dependent mechanisms (30, 31) while suppressing natural killer (NK) cell activity and recruiting regulatory T cells (Tregs) to foster an immunosuppressive niche (32, 33). The marked recurrence risk associated with high TBA (HR=3.00) in our cohort likely stems from such metabolic reprogramming-driven immune evasion and cancer stem cell activation.

MLR, as a marker of inflammatory imbalance, derives its prognostic significance from the functional antagonism between monocytes and lymphocytes. Tumor-associated monocytes polarize into pro-tumor M2 macrophages, secreting IL-10 and TGF-β to inhibit antitumor immunity while facilitating angiogenesis and extracellular matrix remodeling (34, 35). Conversely, lymphocyte depletion (particularly CD8+ T cells) compromises immune surveillance (36). Our finding that elevated MLR (>0.26) independently predicts recurrence (HR=1.66) suggests monocyte-dominated inflammation may trigger NF-κB-mediated epithelial-mesenchymal transition (EMT) and circulating tumor cell (CTC) dissemination, driving early relapse (29, 37). Moreover, monocyte-derived exosomes may transfer oncogenic non-coding RNAs (e.g., miR-21, lncRNA H19) to residual cancer cells, maintaining stemness (38)—a plausible mechanism for the dismal long-term survival in high-risk patients (5-year OS: 19.1%).

We acknowledge that AFP and MVI independently predicted outcomes in multivariate models (**Tables 2**, **3**). However, integrating them into the TBA-MLR score would obscure its core innovation: leveraging metabolic-immune biology for risk stratification. Traditional systems (e.g., BCLC) already incorporate anatomical and tumor-burden variables; our score complements them by identifying high-risk biology within existing stages. For instance, high-risk BCLC 0-A patients (TBA-MLR = 2) had 3.8-fold worse OS than low-risk counterparts (**Supplementary Table S2**), a disparity unexplained by AFP or MVI. Adding AFP/MVI might marginally improve AUC, but at the cost of parsimony and preoperative applicability—contradicting our goal of a simple, mechanism-driven biomarker.

Emerging evidence suggests that the synergistic effect of TBA-MLR scores may stem from metabolic-immune crosstalk. Preclinical studies demonstrate that bile acids activate TGR5 receptors on monocytes and induce immunosuppressive M2 polarization through IL-10 and arginine-1 secretion (27). Conversely, oxidative inflammation-related stress inhibits hepatic CYP7A1 activity, further disrupting bile acid homeostasis (39). This bidirectional interaction accounts for the enhanced predictive accuracy of the combined score compared to individual biomarkers, particularly for long-term prognosis (5-year AUC range: 0.771–0.779). Notably, HCC organoid experiments disclosed that bile acid exposure increased PD-L1 expression while inducing CD8+ T-cell exhaustion, which anti-PD-1 therapy could partially reverse (31). These findings provide some theoretical basis for exploring the potential benefit of immunotherapy in patients with high TBA-MLR scores.

Notably, our subgroup analysis further revealed the clinical significance of this metabolic-immune crosstalk. Patients at high risk (elevated TBA + high MLR) derived no survival benefit from postoperative adjuvant TACE therapy (**Figures 7C, F**). Specifically, their recurrence-free survival (RFS) probability fell below 0.4 within 24 months, and despite intervention, overall survival (OS) approached zero by 48 months. This therapeutic resistance is mechanistically rooted in the biological underpinnings of the score: the high TBA-MLR score reflects both a high systemic tumor burden and an immunosuppressive microenvironment. The excessive systemic tumor burden exceeds the loco-regional control capacity of TACE. At the same time, the immunosuppressive microenvironment blocks TACE-induced immunogenic cell death (ICD) by promoting monocyte polarization towards a pro-tumorigenic M2 phenotype (40, 41). Clinical evidence from the IMbrave 150 trial shows that atezolizumab plus bevacizumab significantly reduces the risk of death by 34% (HR 0.66, 95% CI 0.52–0.85) compared to sorafenib in advanced HCC patients, targeting angiogenesis and immune evasion mechanisms (42). Therefore, high-risk patients may require postoperative adjuvant first-line systemic therapy (e.g., checkpoint inhibitors combined with anti-angiogenic agents) rather than TACE alone.

The clinical implications of our findings are substantial. The TBA-MLR score could be integrated into postoperative decision-making algorithms to identify patients who might benefit from intensified surveillance or adjuvant therapy, even within conventional low-risk groups. For example, a BCLC A patient with a high TBA-MLR score may warrant more frequent imaging (e.g., every 3 months instead of 6) and be considered for enrollment in clinical trials evaluating adjuvant systemic therapies. Conversely, a patient with elevated AFP but a low TBA-MLR score might be spared from overly aggressive interventions. This nuanced approach moves beyond the limitations of rigid staging and towards a more biologically driven, personalized management strategy for HCC.

The novel TBA-MLR scoring system offers three significant advantages over existing approaches: Its cutoff values were objectively determined through maximum rank statistics, overcoming the subjectivity of traditional fixed thresholds and better capturing nonlinear biomarker-outcome relationships. Second, Bootstrap resampling demonstrated that the scoring system was more robust in predicting long-term outcomes (AUC 0.771–0.779 at 5 years) than traditional measures such as AFP (AUC 0.534 at 5 years), consistent with recent research trends on the predictive potential of joint inflammation-metabolism markers (43). Third, and perhaps most clinically significant, the scoring system provides critical prognostic refinement within existing staging systems. It identifies a high-risk subset with dismal outcomes (e.g., 5-year OS of 22.6%) within BCLC stage A, which is traditionally considered low-risk. Most notably, it effectively stratifies risk in the challenging cohort of patients with AFP <400 ng/mL, for whom conventional biomarkers offer little guidance, thereby enabling more personalized postoperative management for these individuals. Notably, BCLC staging relies primarily on anatomical features (e.g., tumor size, vascular invasion) while ignoring the interaction between metabolism and the immune microenvironment. For example, patients in BCLC stage A who had high-risk classifications due to high TBA-MLR scores in this study had a 5-year survival rate of only 15.3%, significantly lower than that of patients in the same stage with low risk (84.7%), suggesting that metabolic-immune disorders may affect prognosis independently of tumor burden. Similarly, AFP as a traditional marker showed a significant decrease in predictive power over time (AUC 0.534 at 5 years), while the TBA-MLR score showed a “time gain effect”(ΔAUC +0.115). This dynamic nature makes it particularly suitable for guiding long-term follow-up strategies, with high-scoring patients likely to require more intensive imaging monitoring and early intervention.

In addition, our comparative survival analyses confirmed that the TBA-MLR score outperforms conventional systems in identifying patients with occult high-risk biology. For example, patients with BCLC 0-A high-risk TBA-MLR score exhibited 5-year OS rates (22.6%) similar to those of the overall cohort’s high-risk group (19.1%), despite being classified as “resectable with good prognosis” under BCLC 0-A staging. This extreme divergence—where 22.6% 5-year OS in TBA-MLR high-risk BCLC 0-A patients contrasts sharply with 86.2% in low-risk counterparts—reveals profound heterogeneity masked by anatomical staging. Such findings highlight that metabolic-immune dysregulation (reflected by elevated TBA and MLR) may drive aggressive tumor behavior independently of tumor size or vascular invasion. Clinically, this supports using the TBA-MLR score to refine postoperative risk assessment, particularly for guiding intensified surveillance (e.g., 3-month imaging for high-risk subgroups) and adjuvant therapy allocation.

Notably, the analyses confirmed minimal concordance between the TBA-MLR score and conventional systems (all |κ|<0.06), with Venn diagrams revealing that 16–25% of high-risk patients identified by our score escaped identification by BCLC or AFP criteria. This independence from tumor-burden variables aligns with the score’s design to capture metabolic-immune dysregulation—a biology not reflected in anatomical staging. Clinically, this enables the TBA-MLR score to uncover high-risk subsets within traditionally “low-risk” groups (e.g., BCLC 0-A), thereby refining postoperative surveillance intensity and adjuvant therapy selection.

Recent advances in artificial intelligence (AI) and machine learning (ML) have significantly advanced the development of prognostic models for HCC. Algorithms such as least absolute shrinkage and selection operator regression (LASSO), bootstrapping, and time-dependent ROC analysis minimize overfitting, enhance feature selection, and improve dynamic risk calibration. The novel TBA-MLR score employs maximally selected rank statistics to determine biomarker cutoffs objectively, overcoming the subjective thresholds inherent in traditional staging systems. Bootstrap validation (1,000 iterations) confirmed its robustness, yielding consistent C-indices for RFS (0.639) and OS (0.682). Compared with established HCC prognostic models—such as the multimodal framework integrating histopathology and serum biomarkers by Zhang et al. (44), and the LASSO-based transplant recurrence nomogram by Gu et al. (45), both requiring complex clinicopathological inputs or tissue analysis—the TBA-MLR score offers superior practicality, relying solely on preoperative blood markers (TBA and MLR). While these nomograms achieve high AUCs (0.739–0.813 at 3 years) through multiparameter integration, our model demonstrates a distinctive time-dependent performance gain (ΔAUC +0.115 over 1–5 years) and enables dynamic risk stratification for long-term monitoring. Future studies integrating AI methodologies—such as deep learning for radiomic feature extraction or neural networks dynamically updating risk scores using longitudinal data—may further enhance precision. For instance, AI could synthesize metabolic-immune markers (e.g., TBA-MLR) with imaging or genomic data to create unified, real-time prognostic platforms.

This study has several limitations that we should acknowledge. Firstly, the single-center, retrospective design inherently limits our study. Although the cohort size (n = 508) provides robust statistical power for internal validation, we exclusively recruited participants from an HBV-endemic region in Southern China (Guangxi), where we observed 70.9% HBsAg-positivity and 37.6% cirrhosis prevalence. This regional specificity may restrict the generalizability of the TBA-MLR score to populations with different etiological profiles (e.g., HCV-driven or alcohol-related HCC in Western cohorts) or genetic backgrounds. Future multicenter prospective studies across diverse geographical settings (e.g., European, North American, and non-HBV Asian cohorts) are imperative to validate the universal applicability of our biomarker. Second, while the baseline TBA and MLR values demonstrate prognostic utility, their dynamic changes during postoperative follow-up remain unclear; future studies incorporating longitudinal data are needed to assess their evolving predictive role. Third, although our model incorporates diverse clinicopathological variables, it does not account for genomic or radiomic features. Integrating multi-omics data (e.g., mutational profiles and imaging biomarkers) could enhance predictive accuracy. Fourth, data on surgical approaches (anatomical vs. non-anatomical resection), adjuvant targeted/immunotherapy, and DCP levels were unavailable. Future studies should incorporate these variables. Finally, to address the limitation of its single-center retrospective origin, a structured validation roadmap is proposed: (1) Multicenter external validation across diverse populations (e.g., HCV-prevalent Western and non-HBV Asian cohorts) via international consortia; (2) Prospective studies tracking post-resection TBA/MLR dynamics for time-dependent risk stratification; (3) Integration with multi-omics data (genomics, radiomics) to enhance accuracy and explore etiology-specific thresholds.

## Conclusion

The TBA-MLR score is a novel and efficient metabolic-immune prognostic tool that effectively identifies high-risk biology occult within conventional HCC staging systems, particularly in BCLC 0-A and AFP <400 ng/mL patients. It enables refined postoperative risk stratification, which provides an essential reference for individualized monitoring and adjuvant treatment strategy. Future multicenter or prospective studies are needed to further validate the value of the score in dynamic monitoring and multidisciplinary treatment decision-making and to explore its synergistic mechanisms with emerging targeted therapies and immunotherapies.

## Data Availability

The raw data supporting the conclusions of this article will be made available by the authors, without undue reservation.

## Supplementary material

Supplementary Figure 1 Kaplan-Meier survival curves comparing RFS and OS among patients stratified by both conventional systems (BCLC stage and AFP level) and the TBA-MLR score. **(A)** RFS stratified by BCLC stage and TBA-MLR score. **(B)** OS stratified by BCLC stage and TBA-MLR score. **(C)** RFS stratified by AFP level and TBA-MLR score. **(D)** OS stratified by AFP level and TBA-MLR score. All comparisons showed statistically significant differences (Log-rank p < 0.0001). The number of patients at risk at specified time points is shown below each panel.

Supplementary Figure 2 Venn diagrams of high-risk patient overlap between TBA-MLR score, BCLC-B, and AFP ≥400 ng/mL for RFS **(A)** and OS **(B)**.

Supplementary Table 1 Stratification of Patients by the TBA-MLR Score within Conventional BCLC and AFP Categories.

Supplementary Table 2 Survival Outcomes Stratified by TBA-MLR Score within BCLC Stage and AFP Categories.

Supplementary Tables 3-4 DeLong’s test results for 1-/3-/5-year RFS and OS comparisons between the TBA-MLR score and other biomarkers (Z-scores and adjusted p-values are provided).

Supplementary Table 5 Baseline Characteristics of HCC Patients Stratified by TBA-MLR Risk Groups for RFS and OS Prediction.

Supplementary Table 6 The Kappa concordance analysis between the TBA-MLR score and BCLC/AFP.
