# Supplementary figures and images for: TBA-MLR score: a metabolic-immune prognostic biomarker for postoperative hepatocellular carcinoma
Source: Front Immunol. 2025 Sep 5;16:1628571. doi: 10.3389/fimmu.2025.1628571 (PMC12446308; doi:10.3389/fimmu.2025.1628571)

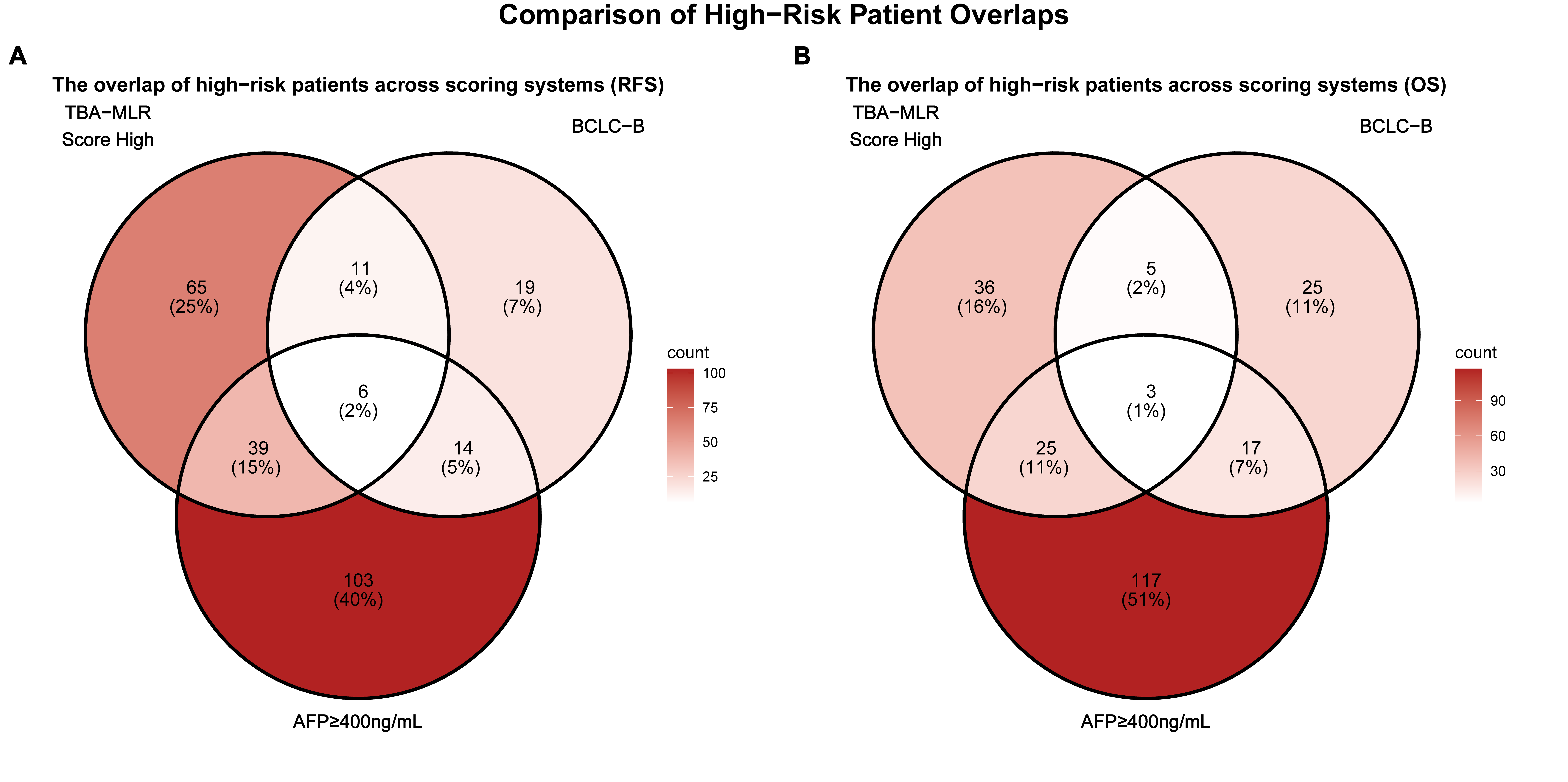

Supplement: Supplementary Figure 2 — Venn diagrams of high-risk patient overlap between TBA-MLR score, BCLC-B, and AFP ≥400 ng/mL for RFS (A) and OS (B). [file Image2.tif]
