# Supplementary material for: TBA-MLR score: a metabolic-immune prognostic biomarker for postoperative hepatocellular carcinoma
Source: Front Immunol. 2025 Sep 5;16:1628571. doi: 10.3389/fimmu.2025.1628571 (PMC12446308; doi:10.3389/fimmu.2025.1628571)
Supplement: Supplementary Table 1 — Stratification of Patients by the TBA-MLR Score within Conventional BCLC and AFP Categories. [file Table1.docx]

****Table S1. Stratification of Patients by the TBA-MLR Score within Conventional BCLC and AFP Categories.****

| ****Variable**** | ****Category**** | ****TBA-MLR Score**** | | |
| --- | --- | --- | --- | --- |
|  |  | ****Low risk**** | ****Intermediate risk**** | ****High risk**** |
| ****RFS**** |  |  |  |  |
| BCLC Stage | **0-A** | 120 (26.2%) | 234 (51.1%) | 104 (22.7%) |
|  | **B** | 5 (10.0%) | 28 (56.0%) | 17 (34.0%) |
| AFP Level | **<400 ng/mL** | 82 (23.6%) | 189 (54.5%) | 76 (21.9%) |
|  | ****≥**400 ng/mL** | 43 (26.7%) | 73 (45.3%) | 45 (28.0%) |
| ****OS**** |  |  |  |  |
| BCLC Stage | **0-A** | 190 (41.5%) | 207 (45.2%) | 61 (13.3%) |
|  | **B** | 10 (20.0%) | 32 (64.0%) | 8 (16.0%) |
| AFP Level | **<400 ng/mL** | 131 (37.8%) | 175 (50.4%) | 41 (11.8%) |
|  | ****≥**400 ng/mL** | 69 (42.9%) | 64 (39.8%) | 28 (17.4%) |
