# Supplementary material for: TBA-MLR score: a metabolic-immune prognostic biomarker for postoperative hepatocellular carcinoma
Source: Front Immunol. 2025 Sep 5;16:1628571. doi: 10.3389/fimmu.2025.1628571 (PMC12446308; doi:10.3389/fimmu.2025.1628571)
Supplement: Supplementary Table 2 — Survival Outcomes Stratified by TBA-MLR Score within BCLC Stage and AFP Categories. [file Table2.docx]

Table S2. Survival Outcomes Stratified by TBA-MLR Score within Traditional BCLC and AFP Categories.

| **Group** | **Recurrence-Free Survival (RFS)** | | | | **Overall Survival (OS)** | | | |
| --- | --- | --- | --- | --- | --- | --- | --- | --- |
|  | **Median (95% CI)** | **1-year RFS** | **3-year RFS** | **5-year RFS** | **Median (95% CI)** | **1-year OS** | **3-year OS** | **5-year OS** |
| BCLC 0-A + Low risk | NR | 95.0% (91.1-99.0) | 82.5% (75.0-90.6) | 75.3% (66.2-85.7) | NR | 98.4% (96.6-100) | 91.8% (87.4-96.5) | 86.2% (80.1-92.9) |
| BCLC 0-A + Intermediate risk | 39.0 months (36.6-50.1) | 80.1% (75.2-85.5) | 58.9% (52.1-66.6) | 36.7% (28.5-47.3) | 97.0 months (68.4-NR) | 92.1% (88.5-95.9) | 74.5% (67.6-82.2) | 60.2% (50.5-71.8) |
| BCLC 0-A+ High risk | 25.8 months (20.0-37.5) | 70.2% (61.9-79.6) | 44.0% (34.9-55.5) | 22.1% (13.6-35.8) | 40.3 months (33.0-NR) | 81.8% (72.6-92.1) | 54.7% (40.2-74.3) | 22.6% (8.4-60.4) |
| BCLC B + Low risk | 35.6 months (NR-NR) | 80.0% (51.6-100) | 0.0% (NR-NR) | 0.0% (NR-NR) | NR (35.6-NR) | 90.0% (73.2-100) | 60.0% (26.3-100) | 60.0% (26.3-100) |
| BCLC B + Intermediate risk | 36.7 months (16.6-NR) | 78.3% (64.3-95.3) | 54.6% (35.5-84.0) | 13.6% (2.4-78.6) | NR (50.8-NR) | 93.8% (85.7-100) | 89.8% (79.5-100) | 59.9% (26.7-100) |
| BCLC B + High risk | 10.2 months (4.4-NR) | 47.1% (28.4-77.9) | 24.7% (9.9-61.6) | 12.4% (2.3-65.0) | 19.9 months (4.9-NR) | 62.5% (36.5-100) | 25.0% (7.5-83.0) | 0.0% (NR-NR) |
| AFP Low + Low risk | NR | 93.9% (88.8-99.2) | 79.6% (70.0-90.4) | 71.2% (59.4-85.2) | NR | 97.7% (95.1-100) | 87.9% (81.5-94.7) | 82.7% (74.8-91.6) |
| AFP Low + Intermediate risk | 39.8 months (36.9-66.7) | 83.4% (78.2-88.9) | 61.4% (53.9-69.9) | 41.6% (32.3-53.5) | 97.0 months (83.1-NR) | 97.6% (95.4-100) | 81.8% (74.7-89.5) | 67.1% (56.0-80.4) |
| AFP Low + High risk | 36.3 months (25.6-45.3) | 80.3% (71.8-89.7) | 50.5% (39.6-64.4) | 29.0% (18.3-46.2) | 45.5 months (34.1-NR) | 87.5% (77.9-98.4) | 63.8% (47.5-85.8) | 31.9% (12.4-81.9) |
| AFP High + Low risk | NR (74.4-NR) | 95.3% (89.1-100) | 83.1% (71.4-96.7) | 78.5% (65.0-94.8) | NR (87.6-NR) | 98.5% (95.5-100) | 96.8% (92.6-100) | 90.5% (81.4-100) |
| AFP High + Intermediate risk | 36.3 months (29.6-47.1) | 70.9% (61.2-82.2) | 51.2% (39.3-66.7) | 16.6% (6.8-41.0) | 49.3 months (23.5-NR) | 77.5% (67.8-88.7) | 58.8% (45.8-75.5) | 42.7% (27.5-66.4) |
| AFP High + High risk | 7.8 months (5.3-20.0) | 44.4% (32.1-61.6) | 26.0% (15.7-43.0) | 0.0% (NR-NR) | 30.0 months (14.2-NR) | 67.9% (52.6-87.6) | 34.7% (18.3-65.6) | 0.0% (NR-NR) |

NR: Not Reached (median survival not achieved); HR: Hazard Ratio.
