# Supplementary material for: TBA-MLR score: a metabolic-immune prognostic biomarker for postoperative hepatocellular carcinoma
Source: Front Immunol. 2025 Sep 5;16:1628571. doi: 10.3389/fimmu.2025.1628571 (PMC12446308; doi:10.3389/fimmu.2025.1628571)
Supplement: Supplementary Tables 3-4 — DeLong’s test results for 1-/3-/5-year RFS and OS comparisons between the TBA-MLR score and other biomarkers (Z-scores and adjusted p-values are provided). [file Table3.docx]

**Table S3.** DeLong test results of TBA-MLR score predicting RFS versus other indicators.

| **Time（months）** | ****Comparator**** | ****Z-score**** | ****P-value (original/adjusted)**** |
| --- | --- | --- | --- |
| **12** | **MLR** | **7.35** | **1.91e-13 / 1.72e-12** |
| **12** | **SIRI** | **6.57** | **4.93e-11 / 2.22e-10** |
| **12** | **SII** | **5.14** | **2.78e-07 / 8.34e-07** |
| **12** | **PLR** | **4.93** | **8.22e-07 / 1.85e-06** |
| **12** | **NLR** | **4.70** | **2.59e-06 / 4.66e-06** |
| **12** | **Child-Pugh** | **4.57** | **4.79e-06 / 7.18e-06** |
| **12** | **BCLC** | **3.10** | **0.0019 / 0.0025** |
| **12** | **AFP** | **2.58** | **0.0099 / 0.0111** |
| **12** | **TBA** | **-1.09** | **0.2742 / 0.2742** |
| **36** | **MLR** | **7.01** | **2.36e-12 / 2.13e-11** |
| **36** | **SIRI** | **6.09** | **1.11e-09 / 4.99e-09** |
| **36** | **SII** | **5.86** | **4.70e-09 / 1.41e-08** |
| **36** | **Child-Pugh** | **5.77** | **8.15e-09 / 1.83e-08** |
| **36** | **PLR** | **5.10** | **3.44e-07 / 6.19e-07** |
| **36** | **NLR** | **4.48** | **7.42e-06 / 1.11e-05** |
| **36** | **BCLC** | **3.78** | **0.0002 / 0.0002** |
| **36** | **AFP** | **2.77** | **0.0057 / 0.0064** |
| **36** | **TBA** | **-1.67** | **0.0947 / 0.0947** |
| **60** | **MLR** | **8.79** | **1.56e-18 / 1.41e-17** |
| **60** | **SIRI** | **6.92** | **4.50e-12 / 2.03e-11** |
| **60** | **Child-Pugh** | **6.03** | **1.65e-09 / 4.95e-09** |
| **60** | **SII** | **5.95** | **2.70e-09 / 6.09e-09** |
| **60** | **PLR** | **5.48** | **4.35e-08 / 7.83e-08** |
| **60** | **NLR** | **4.48** | **7.32e-06 / 1.10e-05** |
| **60** | **BCLC** | **3.75** | **0.0002 / 0.0002** |
| **60** | **TBA** | **-3.05** | **0.0023 / 0.0026** |
| **60** | **AFP** | **2.82** | **0.0047 / 0.0047** |
