# Supplementary material for: TBA-MLR score: a metabolic-immune prognostic biomarker for postoperative hepatocellular carcinoma
Source: Front Immunol. 2025 Sep 5;16:1628571. doi: 10.3389/fimmu.2025.1628571 (PMC12446308; doi:10.3389/fimmu.2025.1628571)
Supplement: Supplementary file 6 [file Table4.docx]

**Table S4.** DeLong test results for TBA-MLR score predicting OS versus other measures.

| **Time（months）** | ****Comparator**** | ****Z-score**** | ****P-value (original/adjusted)**** |
| --- | --- | --- | --- |
| **12** | **MLR** | 3.75 | 0.0002/0.0016 |
| **12** | **SIRI** | 2.71 | 0.0067/0.0278 |
| **12** | **SII** | 2.60 | 0.0093/0.0278 |
| **12** | **PLR** | 2.33 | 0.0198/0.0427 |
| **12** | **NLR** | 2.26 | 0.0237/0.0427 |
| **12** | **Child-Pugh** | 2.16 | 0.0304/0.0457 |
| **12** | **BCLC** | 1.94 | 0.0519/0.0667 |
| **12** | **AFP** | 1.28 | 0.2018/0.2271 |
| **12** | **TBA** | -0.82 | 0.4140/0.4140 |
| **36** | **MLR** | 6.46 | 1.02e-10/9.20e-10 |
| **36** | **SIRI** | 4.12 | 3.75e-05/1.70e-04 |
| **36** | **SII** | 3.97 | 7.26e-05/2.20e-04 |
| **36** | **Child-Pugh** | 3.49 | 0.0005/0.0011 |
| **36** | **PLR** | 3.27 | 0.0011/0.0019 |
| **36** | **NLR** | 2.80 | 0.0052/0.0078 |
| **36** | **BCLC** | -2.64 | 0.0084/0.0105 |
| **36** | **AFP** | 2.60 | 0.0093/0.0105 |
| **36** | **TBA** | 1.22 | 0.2209/0.2209 |
| **60** | **MLR** | 7.53 | 5.13e-14/4.62e-13 |
| **60** | **SIRI** | 5.38 | 7.54e-08/3.39e-07 |
| **60** | **Child-Pugh** | 4.65 | 3.28e-06/9.83e-06 |
| **60** | **SII** | 4.32 | 1.55e-05/3.48e-05 |
| **60** | **PLR** | 3.85 | 0.0001/0.0002 |
| **60** | **NLR** | 3.47 | 0.0005/0.0008 |
| **60** | **BCLC** | 3.06 | 0.0022/0.0028 |
| **60** | **TBA** | -2.89 | 0.0039/0.0044 |
| **60** | **AFP** | 1.72 | 0.0848/0.0848 |
