# Supplementary material for: TBA-MLR score: a metabolic-immune prognostic biomarker for postoperative hepatocellular carcinoma
Source: Front Immunol. 2025 Sep 5;16:1628571. doi: 10.3389/fimmu.2025.1628571 (PMC12446308; doi:10.3389/fimmu.2025.1628571)
Supplement: Supplementary Table 5 — Baseline Characteristics of HCC Patients Stratified by TBA-MLR Risk Groups for RFS and OS Prediction. [file Table5.docx]

**Table S5. Baseline Characteristics of HCC Patients Stratified by TBA-MLR Risk Groups for RFS and OS Prediction.**

| ****Characteristic**** | **RFS Risk Stratification** | | | |  | ****OS Risk Stratification**** | | | |
| --- | --- | --- | --- | --- | --- | --- | --- | --- | --- |
|  | ****High risk (n=121)**** | ****Intermediate (n=262)**** | ****Low risk (n=125)**** | ****p-value**** |  | ****High risk (n=69)**** | ****Intermediate (n=239)**** | ****Low risk (n=200)**** | ****p-value**** |
| **Gender, n (%)** |  |  |  | **0.051** |  |  |  |  | **0.092** |
| Female | **14 (11.6%)** | **52 (19.8%)** | **29 (23.2%)** |  |  | **7 (10.1%)** | **44 (18.4%)** | **44(22.0%)** |  |
| Male | **107 (88.4%)** | **210 (80.2%)** | **96 (76.8%)** |  |  | **62 (89.9%)** | **195 (81.6%)** | **156(78.0%)** |  |
| **Age (years), n (%)** |  |  |  | **0.207** |  |  |  |  | **0.102** |
| **＜60** | **76 (62.8%)** | **171 (65.3%)** | **91 (72.8%)** |  |  | **47 (68.1%)** | **148 (61.9%)** | **143 (71.5%)** |  |
| **≥60** | **45 (37.2%)** | **91 (34.7%)** | **34 (27.2%)** |  |  | **22 (31.9%)** | **91 (38.1%)** | **57 (28.5%)** |  |
| **BMI (kg/m^2^), n (%)** |  |  |  | **0.981** |  |  |  |  | **0.536** |
| **<18.5** | **7 ( 5.8%)** | **139 (53.1%)** | **91 (72.8%)** |  |  | **6 ( 8.7%)** | **12 (5.0%)** | **9 (4.5%)** |  |
| **18.5–24** | **61 (50.4%)** | **13 ( 5.0%)** | **55 (44.0%)** |  |  | **34 (49.3%)** | **130 ( 54.4%)** | **99 (49.5%)** |  |
| **＞24** | **53 (43.8%)** | **110 (42.0%)** | **55 (44.0%)** |  |  | **29 (42.0%)** | **97 (40.6%)** | **92 (46.0%)** |  |
| **CRP (mg/L), n (%)** |  |  |  |  |  |  |  |  | **0.718** |
| **≤10** | **69 (57.0%)** | **141 (53.8%)** | **68 (54.4%)** | **0.839** |  | **39 (56.5%)** | **134 (56.1%)** | **105 (52.5%)** |  |
| **＞10** | **52 (43.0%)** | **121 (46.2%)** | **57 (45.6%)** |  |  | **30 (43.5%)** | **105 (43.9%)** | **95 (47.5%)** |  |
| **PLT (×10⁹/L), n (%)** |  |  |  | **0.099** |  |  |  |  | ****0.035**** |
| **<100** | **16 (13.2%)** | **23 ( 8.8%)** | **13 (10.4%)** |  |  | **11 (15.9%)** | **21 ( 8.8%)** | **20 (10.0%)** |  |
| **100–300** | **86 (71.1%)** | **218 (83.2%)** | **98 (78.4%)** |  |  | **45 (65.2%)** | **198(82.8%)** | **159 (79.5%)** |  |
| **>300** | **19 (15.7%)** | **21 ( 8.0%)** | **14 (11.2%)** |  |  | **13 (18.8%)** | **20 ( 8.4%)** | **21 (10.5%)** |  |
| **ALT(U/L), n (%)** |  |  |  |  |  |  |  |  | **0.340** |
| **≤50** | **86 (71.1%)** | **198 (75.6%)** | **97 (77.6%)** | **0.475** |  | **48 (69.6%)** | **177(74.1%)** | **156 (78.0%)** |  |
| **＞50** | **35 (28.9%)** | **64 (24.4%)** | **28 (22.4%)** |  |  | **21 (30.4%)** | **62 (25.9%)** | **44 (22.0%)** |  |
| **AST(U/L), n (%)** |  |  |  | ****0.019**** |  |  |  |  | ****0.001**** |
| **≤40** | **61 (50.4%)** | **154 (58.8%)** | **85 (68.0%)** |  |  | **33 (47.8%)** | **130 (54.4%)** | **137 (68.5%)** |  |
| **＞40** | **60 (49.6%)** | **108 (41.2%)** | **40 (32.0%)** |  |  | **36 (52.2%)** | **109 (45.6%)** | **63 (31.5%)** |  |
| **TBIL (µmol/L), n (%)** |  |  |  | ****0.005**** |  |  |  |  | ****＜0.001**** |
| **≤17.1** | **74 (61.2%)** | **188 (71.8%)** | **100 (80.0%)** |  |  | **40 (58.0%)** | **160 (66.9%)** | **162 (81.0%)** |  |
| **>17.1** | **47 (38.8%)** | **74 (28.2%)** | **25 (20.0%)** |  |  | **29 (42.0%)** | **79 (33.1%)** | **38 (19.0%)** |  |
| **ALB (g/L), n (%)** |  |  |  | ****0.004**** |  |  |  |  | ****0.003**** |
| **<35** | **42 (34.7%)** | **61 (23.3%)** | **21 (16.8%)** |  |  | **23 (33.3%)** | **68 (28.5%)** | **33 (16.5%)** |  |
| **≥35** | **79 (65.3%)** | **201 (76.7%)** | **104 (83.2%)** |  |  | **46 (66.7%)** | **171 (71.5%)** | **167 (83.5%)** |  |
| **AFP (ng/mL), n (%)** |  |  |  | ****0.044**** |  |  |  |  | **NA**† |
| **≤200** | **64 (52.9%)** | **169 (64.5%)** | **75 (60.0%)** |  |  | **36 (52.2%)** | **155 (64.9%)** | **117 (58.5%)** |  |
| **200-1000** | **52 (43.0%)** | **83 (31.7%)** | **39 (31.2%)** |  |  | **29 (42.0%)** | **76 (31.8%)** | **69 (34.5%)** |  |
| **>1000** | **5 ( 4.1%)** | **10 ( 3.8%)** | **11 ( 8.8%)** |  |  | **4 ( 5.8%)** | **8 ( 3.3%)** | **14 ( 7.0%)** |  |
| **CEA (ng/mL), n (%)** |  |  |  |  |  |  |  |  | **0.756** |
| **≤5** | **113 (93.4%)** | **244 (93.1%)** | **121 (96.8%)** | **0.334** |  | **65 (94.2%)** | **223 (93.3%)** | **190 (95.0%)** |  |
| **>5** | **8 ( 6.6%)** | **18 ( 6.9%)** | **4 ( 3.2%)** |  |  | **4 ( 5.8%)** | **16 ( 6.7%)** | **10 ( 5.0%)** |  |
| **Cirrhosis, n (%)** |  |  |  | ****<0.001**** |  |  |  |  | ****0.001**** |
| **no** | **57 (47.1%)** | **171 (65.3%)** | **89 (71.2%)** |  |  | **31 (44.9%)** | **147 (61.5%)** | **139 (69.5%)** |  |
| **yes** | **64 (52.9%)** | **91 (34.7%)** | **36 (28.8%)** |  |  | **38 (55.1%)** | **92 (38.5%)** | **61 (30.5%)** |  |
| HBsAg**, n (%)** |  |  |  | **0.420** |  |  |  |  | **0.353** |
| **negative** | **35 (28.9%)** | **82 (31.3%)** | **31 (24.8%)** |  |  | **18 (26.1%)** | **77 (32.2%)** | **53 (26.5%)** |  |
| **positive** | **86 (71.1%)** | **180 (68.7%)** | **94 (75.2%)** |  |  | **51 (73.9%)** | **162 (67.8%)** | **147 (73.5%)** |  |
| HBV-DNA(IU/mL)**, n (%)** |  |  |  | **0.927** |  |  |  |  | **0.995** |
| **＜500** | **78 (64.5%)** | **164 (62.6%)** | **80 (64.0%)** |  |  | **44 (63.8%)** | **151 (63.2%)** | **127 (63.5%)** |  |
| **≥500** | **43 (35.5%)** | **98 (37.4%)** | **45 (36.0%)** |  |  | **25 (36.2%)** | **88 (36.8%)** | **73 (36.5%)** |  |
| Child-Pugh class**, n (%)** |  |  |  | ****0.012**** |  |  |  |  | ****0.004**** |
| **A** | **105 (86.8%)** | **243 (92.7%)** | **121 (96.8%)** |  |  | **60 (87.0%)** | **215 (90.0%)** | **194 (97.0%)** |  |
| **B** | **16 (13.2%)** | **19 ( 7.3%)** | **4 ( 3.2%)** |  |  | **9 (13.0%)** | **24 (10.0%)** | **6 (3.0%)** |  |
| Tumor number**, n (%)** |  |  |  | ****0.008**** |  |  |  |  | ****0.003**** |
| **Single** | **97 (80.2%)** | **223 (85.1%)** | **117 (93.6%)** |  |  | **55 (79.7%)** | **197 (82.4%)** | **185 (92.5%)** |  |
| **Multiple** | **24 (19.8%)** | **39 (14.9%)** | **8 ( 6.4%)** |  |  | **14 (20.3%)** | **42 (17.6%)** | **15 (7.5%)** |  |
| **Tumor size (cm), n (%)** |  |  |  | **0.104** |  |  |  |  | **0.139** |
| **<5** | **58 (47.9%)** | **150 (57.3%)** | **76 (60.8%)** |  |  | **32 (46.4%)** | **132 (55.2%)** | **120 (60.0%)** |  |
| **≥5** | **63 (52.1%)** | **112 (42.7%)** | **49 (39.2%)** |  |  | **37 (53.6%)** | **107 (44.8%)** | **80 (40.0%)** |  |
| **MVI, n (%)** |  |  |  | ****0.032**** |  |  |  |  | ****0.010**** |
| **no** | **73 (60.3%)** | **168 (64.1%)** | **94 (75.2%)** |  |  | **39 (56.5%)** | **149 (62.3%)** | **147 (73.5%)** |  |
| **yes** | **48 (39.7%)** | **94 (35.9%)** | **31 (24.8%)** |  |  | **30 (43.5%)** | **90 (37.7%)** | **53 (26.5%)** |  |
| Differentiation**, n (%)** |  |  |  | **NA**† |  |  |  |  | **NA**† |
| **PD** | **4 ( 3.3%)** | **23 ( 8.8%)** | **12 ( 9.6%)** |  |  | **3 (4.3%)** | **15 (6.3%)** | **21 (10.5%)** |  |
| **MD** | **102 (84.3%)** | **207 (79.0%)** | **104 (83.2%)** |  |  | **61 (88.4%)** | **190 (79.5%)** | **162 (81.0%)** |  |
| **WD** | **15 (12.4%)** | **32 (12.2%)** | **9 ( 7.2%)** |  |  | **5 (7.2%)** | **34 (14.2%)** | **17 (8.5%)** |  |
| Hepatic capsule invasion**, n (%)** |  |  |  | **0.802** |  |  |  |  | **0.247** |
| **no** | **95 (78.5%)** | **204 (77.9%)** | **101 (80.8%)** |  |  | **53 (76.8%)** | **182 (76.2%)** | **165 (82.5%)** |  |
| **yes** | **26 (21.5%)** | **58 (22.1%)** | **24 (19.2%)** |  |  | **16 (23.2%)** | **57 (23.8%)** | **35 (17.5%)** |  |
| BCLC**, n (%)** |  |  |  | **NA**† |  |  |  |  | ****0.041**** |
| **0** | **9 ( 7.4%)** | **21 ( 8.0%)** | **11 ( 8.8%)** |  |  | **6 (8.7%)** | **17 (7.1%)** | **18 (9.0%)** |  |
| **A** | **95 (78.5%)** | **213 (81.3%)** | **109 (87.2%)** |  |  | **55 (79.7%)** | **190 (79.5%)** | **172 (86.0%)** |  |
| **B** | **17 (14.0%)** | **28 (10.7%)** | **5 ( 4.0%)** |  |  | **8 (11.6%)** | **32 (13.4%)** | **10 (5.0%)** |  |
| TACE**, n (%)** |  |  |  | ****<0.001**** |  |  |  |  | ****＜0.001**** |
| **no** | **84 (69.4%)** | **197 (75.2%)** | **113 (90.4%)** |  |  | **47 (68.1%)** | **174 (72.8%)** | **173 (86.5%)** |  |
| **yes** | **37 (30.6%)** | **65 (24.8%)** | **12 ( 9.6%)** |  |  | **22 (31.9%)** | **65 (27.2%)** | **27 (13.5%)** |  |
| Recurrence mode**, n (%)** |  |  |  | **NA**† |  |  |  |  | **NA**† |
| **NR** | **39 (32.2%)** | **144 (55.0%)** | **100 (80.0%)** |  |  | **24 (34.8%)** | **122 (51.0%)** | **137 (68.5%)** |  |
| **IHR** | **53 (43.8%)** | **78 (29.8%)** | **23 (18.4%)** |  |  | **26 (37.7%)** | **74 (31.0%)** | **54 (27.0%)** |  |
| **PM** | **19 (15.7%)** | **20 ( 7.6%)** | **1 ( 0.8%)** |  |  | **16 (23.2%)** | **21 (8.8%)** | **3 (1.5%)** |  |
| NP-EHM | **10 ( 8.3%)** | **20 ( 7.6%)** | **1 ( 0.8%)** |  |  | **3 (4.3%)** | **22 (9.2%)** | **6 (3.0%)** |  |

**Abbreviations: BMI, body mass index; CRP, C-reactive protein; MLR, monocyte-to-lymphocyte ratio; PLT, platelet count; TBA, total bile acid; ALT, alanine aminotransferase; AST, aspartate aminotransferase; TBIL, total bilirubin; ALB, albumin; AFP, alpha-fetoprotein; CEA, carcinoembryonic antigen; HBsAg, hepatitis B surface antigen; MVI, microvascular invasion;** PD, Poorly differentiated; MD, Moderately differentiated; WD, Well-differentiated; **BCLC, Barcelona Clinic Liver Cancer;** TACE, Transcatheter Arterial Chemoembolization; IHR, Intrahepatic Recurrence; PM, Pulmonary Metastasis; NP-EHM, Non-Pulmonary Extrahepatic Metastases; NR, No Recurrence.

†Categorical variables were analyzed using Fisher's exact test, with NA values denoting variables where test assumptions were violated (e.g., sparse data such as <5 cases in the poorly differentiated tumor subgroup of low-risk patients).
