# Supplementary material for: TBA-MLR score: a metabolic-immune prognostic biomarker for postoperative hepatocellular carcinoma
Source: Front Immunol. 2025 Sep 5;16:1628571. doi: 10.3389/fimmu.2025.1628571 (PMC12446308; doi:10.3389/fimmu.2025.1628571)
Supplement: Supplementary Table 6 — The Kappa concordance analysis between the TBA-MLR score and BCLC/AFP. [file Table6.docx]

**Table S6.  The Kappa concordance analysis between TBA-MLR Score and BCLC/AFP.**

| Group | Comparison | Kappa Type | κ | 95% CI | z | p |
| --- | --- | --- | --- | --- | --- | --- |
| FRS | TBA-MLR vs BCLC | Unweighted | 0.015 | (-0.042, 0.071) | 0.504 | 0.615 |
|  |  | Weighted | 0.041 | (-0.016, 0.098) | 1.421 | 0.155 |
| FRS | TBA-MLR vs AFP | Unweighted | -0.037 | (-0.088, 0.013) | -1.460 | 0.146 |
| OS | TBA-MLR vs BCLC | Unweighted | -0.011 | (-0.061, 0.039) | -0.421 | 0.674 |
|  |  | Weighted | 0.020 | (-0.030, 0.069) | 0.776 | 0.438 |
| OS | TBA-MLR vs AFP | Unweighted | -0.056 | (-0.120, 0.009) | -1.690 | 0.091 |

Notes:

1. κ = Cohen's kappa coefficient.

2. All kappa values indicate slight agreement (|κ| < 0.20) according to Landis and Koch criteria.

3. Sample size n = 508 for all comparisons.

4. No statistically significant agreement found (all p-values > 0.05).
